# ACE2-EGFR-MAPK signaling contributes to SARS-CoV-2 infection

**DOI:** 10.26508/lsa.202201880

**Published:** 2023-07-04

**Authors:** Melanie Engler, Dan Albers, Pascal Von Maltitz, Rüdiger Groß, Jan Münch, Ion Cristian Cirstea

**Affiliations:** 1 Institute of Comparative Molecular Endocrinology, Ulm University , Ulm, Germany; 2 Institute of Molecular Virology, Ulm University Medical Center, Ulm, Germany

## Abstract

SARS-CoV-2 triggered the most severe pandemic of recent times. To enter into a host cell, SARS-CoV-2 binds to the angiotensin-converting enzyme 2 (ACE2). However, subsequent studies indicated that other cell membrane receptors may act as virus-binding partners. Among these receptors, the epidermal growth factor receptor (EGFR) was hypothesized not only as a spike protein binder, but also to be activated in response to SARS-CoV-2. In our study, we aim at dissecting EGFR activation and its major downstream signaling pathway, the mitogen-activated signaling pathway (MAPK), in SARS-CoV-2 infection. Here, we demonstrate the activation of EGFR–MAPK signaling axis by the SARS-CoV-2 spike protein and we identify a yet unknown cross talk between ACE2 and EGFR that regulated ACE2 abundance and EGFR activation and subcellular localization, respectively. By inhibiting the EGFR-MAPK activation, we observe a reduced infection with either spike-pseudotyped particles or authentic SARS-CoV-2, thus indicating that EGFR serves as a cofactor and the activation of EGFR-MAPK contributes to SARS-CoV-2 infection.

## Abstract (precis)

SARS-CoV-2 activates the conserved EGFR-RAS-MAPK pathway through a novel mechanism that involves an ACE2-EGFR cross talk and pharmacological inhibition of the MAPK pathway is able to reduce viral infection.

## Graphical Abstract

**Figure:**
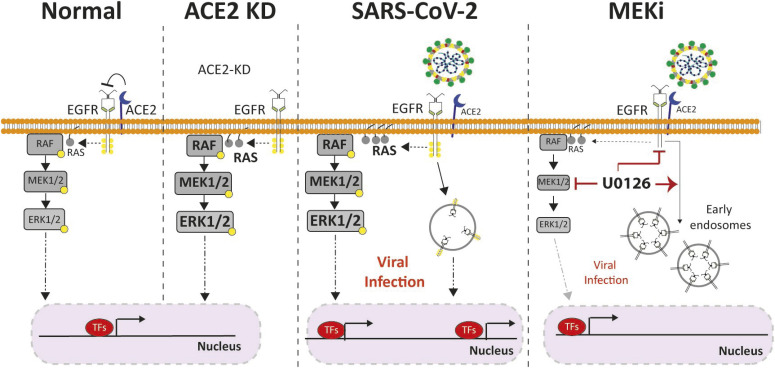

## Introduction

Infectious disease outbreaks reaching a pandemic scale are a continuous challenge for public health in a globalized world, with the SARS-CoV-2 pandemic reaching a scale that was not seen since the Spanish flu outbreak. Ever since the first case was reported in 2019 in Wuhan (China) (Zhou et al, 2020), coronavirus disease-19 (COVID-19) caused millions of deaths worldwide and still presents a global challenge (Wang et al, 2022). SARS-CoV-2 belongs to the genus Betacoronavirus (Lu et al, 2020) and harbors specific glycoproteins in the viral envelope, termed spike proteins, which mediate binding to and infection of the host cell. The spike protein consists of two subunits, S1 and S2 (Spiga et al, 2003). S1 contains the receptor-binding domain (RBD) that binds to the angiotensin-converting enzyme 2 (ACE2), which serves as main receptor for SARS-CoV-2 (Shang et al, 2020a; Lan et al, 2020). S2 is activated by proteolytic cleavage between S1 and S2 and initiates the fusion of the viral envelope with the host cell membrane (Bestle et al, 2020; Shang et al, 2020b). This proteolytic activation of the spike protein can either happen at the cell membrane, by the transmembrane protease serine subtype 2 (TMPRSS2), or in the endolysosomes, after clathrin-dependent endocytosis of SARS-CoV-2, by cathepsins (Bestle et al, 2020; Bayati et al, 2021).

Although ACE2 serves as the main receptor, ACE2-independent mechanisms were shown in T lymphocytes (Shen et al, 2022) and hepatocytes (Wanner et al, 2022). In addition, the E484D mutation of the spike protein demonstrated an ACE2-independent entry in Huh7 cells (Hoffmann et al, 2022) and the lung cell line H522 (Puray-Chavez et al, 2021), challenging the dogma of ACE2 being the only receptor interacting with SARS-CoV-2.

Indeed, several SARS-CoV-2 co-receptors have been proposed like Neuropilin 1 (NRP1) (Cantuti-Castelvetri et al, 2020) and the tyrosine-protein kinase receptor UFO (AXL) (Wang et al, 2021) among others. Although ACE2 serves as the main receptor, a recent affinity study identified the epidermal growth factor receptor (EGFR), besides others, as a potential receptor candidate for SARS-CoV-2 (Wang et al, 2021). In addition, a leverage docking analysis between the SARS-CoV-2 spike protein and EGFR using glioma cells revealed a similar binding affinity as the one between spike and ACE2 (Khan & Hatiboglu, 2020). Proteomic and bioinformatics studies in Caco-2 and A549 cells expressing ACE2 (A549-ACE2 cells) that were infected with SARS-CoV-2 suggested increased EGFR signaling (Klann et al, 2020; Stukalov et al, 2021). In addition to several *omic* studies, a recently published study described an increased EGFR phosphorylation in response to the full-length spike protein using A549 cells that are characterized by low levels of endogenous ACE2 (Palakkott et al, 2023). Therefore, EGFR could be a promising candidate of a co-receptor to ACE2 for SARS-CoV-2 infection.

EGFR belongs to the class of receptor tyrosine kinases, cell surface receptors with wide-tissue and cell-type distribution. EGFR can be activated by various ligands, including EGF, TGF-α, amphiregulin, epiregulin, HB-EGF, betacellulin, and epigen (Schneider & Wolf, 2009). Its major signaling axis is represented by the canonical RAS-MAPK pathway, a crucial regulator of biological processes such as proliferation, differentiation, survival, and migration (Braicu et al, 2019). Upon ligand binding and activation, EGFR recruits transducer molecules that lead to the activation of rat sarcoma (RAS) GTPase, which in turn activates rapid accelerated fibrosarcoma 1 (CRAF)—MAPKs 1/2 (MEK1/2)—extracellular signal-regulated kinase (ERK1/2) kinase cascade. However, there are also ligand/kinase-independent (noncanonical) modes of action of EGFR described for the regulation of autophagy and metabolism, among other processes (Tan et al, 2015, 2016).

That the EGFR-MAPK signaling axis is implicated in SARS-CoV-2 infection was demonstrated by the antiviral properties of EGFR and MEK1/2 inhibitors (Chen et al, 2020; Schreiber et al, 2022). In addition, increased EGFR levels were detected in the lung tissue of SARS-CoV-2 deceased individuals (e.g., type I and type II pneumocytes, alveolar macrophages, and fibroblasts), and EGFR was identified as a key regulator of fibrosis in COVID-19 (Vagapova et al, 2021) suggesting the use of anti-EGFR antibodies to reduce inflammation and lung fibrosis in severe COVID-19 patients (Londres et al, 2022). However, not much is known about the function of EGFR during SARS-CoV-2 infection. Thus, we here characterized the activation and cellular effects of EGFR–RAS–MAPK signaling pathways in response to SARS-CoV-2 infection of Caco-2 cells. Taken together, our results demonstrate the activation of the RAS–MAPK pathway in response to spike protein binding, that EGFR is a novel cofactor of ACE2 that promotes SARS-CoV-2 entry and that inhibition has potential to reduce viral infection.

## Results

### SARS-CoV-2 Spike-RBD activates EGFR and MAPK

**Figure S1.**
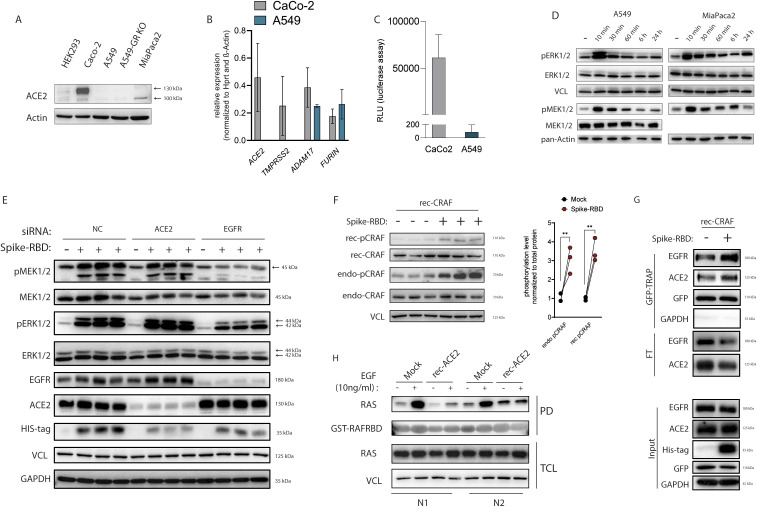
Spike-receptor-binding domain (RBD) effects on MAPK activation in A549 and MiaPaca2 cell lines and the role of angiotensin-converting enzyme 2 (ACE2) as a negative regulator of epidermal growth factor receptor (EGFR)-MAPK signaling. **(A)** Immunoblot of endogenous ACE2 in total protein lysates from HEK293T, Caco-2, A549, A549 lacking the gene for the glucocorticoid receptor (GR KO) and MiaPaca2 cells. **(B)** mRNA transcripts relative to housekeeping genes of ACE2 and proteases involved in Spike protein cleavage in A549 and Caco-2 cells as measured using qRT-PCR. **(C)** Caco-2 and A549 cells were infected with VSV-based viral particles pseudotyped with SARS-CoV-2-Spike (Spike-PP) and cell entry was determined by measuring luciferase activity after 24 h. **(D)** Activation of ERK1/2 and MEK1/2 was determined by detecting phosphorylation level of ERK1/2 and MEK1/2 by immunoblotting in A549 and MiaPaca2 cells treated with 100 ng of Spike-RBD for indicated time points. ERK1/2 and MEK1/2 blots were used as control for their phospho-forms, whereas vinculin and pan-actin were used as loading control. **(E)** MAPK activation in ACE2 and EGFR siRNA-mediated knockdown (KD) Caco-2 cells after 10 min of Spike-RBD incubation. Scramble siRNA was used as negative control (NC). Target proteins were detected by immunoblotting using specific antibodies. MEK1/2 and ERK1/2 blots were used as control for their phospho-forms, whereas EGFR and ACE2 serve as KD controls, and GAPDH and vinculin were used as loading controls. **(F, G)** Caco-2 cells transfected with EGFP-CRAF plasmid were treated with 100 ng Spike-RBD for 10 min. **(F)** Spike-RBD-induced activation of endogenous CRAF (endo-CRAF) and recombinant CRAF (rec-CRAF) was detected by immunoblotting using specific antibody against activating phosphorylation at Ser338 of CRAF. **(G)** Spike-RBD-induced complex formation of EGFP-CRAF with EGFR and ACE2 in response to Spike-RBD treatment for 10 min was analyzed by subjecting Caco-2 cells expressing EGFP-rec-CRAF to GFP-TRAP assay. Protein levels were determined by immunoblotting in the input, flow-through (FT), and GFP-TRAP–precipitated fraction (GFP-TRAP). **(H)** HEK293T cells were transfected with human ACE2 (rec-ACE2) and stimulated with 10 ng/ml EGF for 10 min. Total cell lysates were used for pull down (PD) assay using GST-CRAF-RBD as bait, followed by immunoblotting, using RAS antibody to detect active GTP-bound RAS.

**Figure 1.**
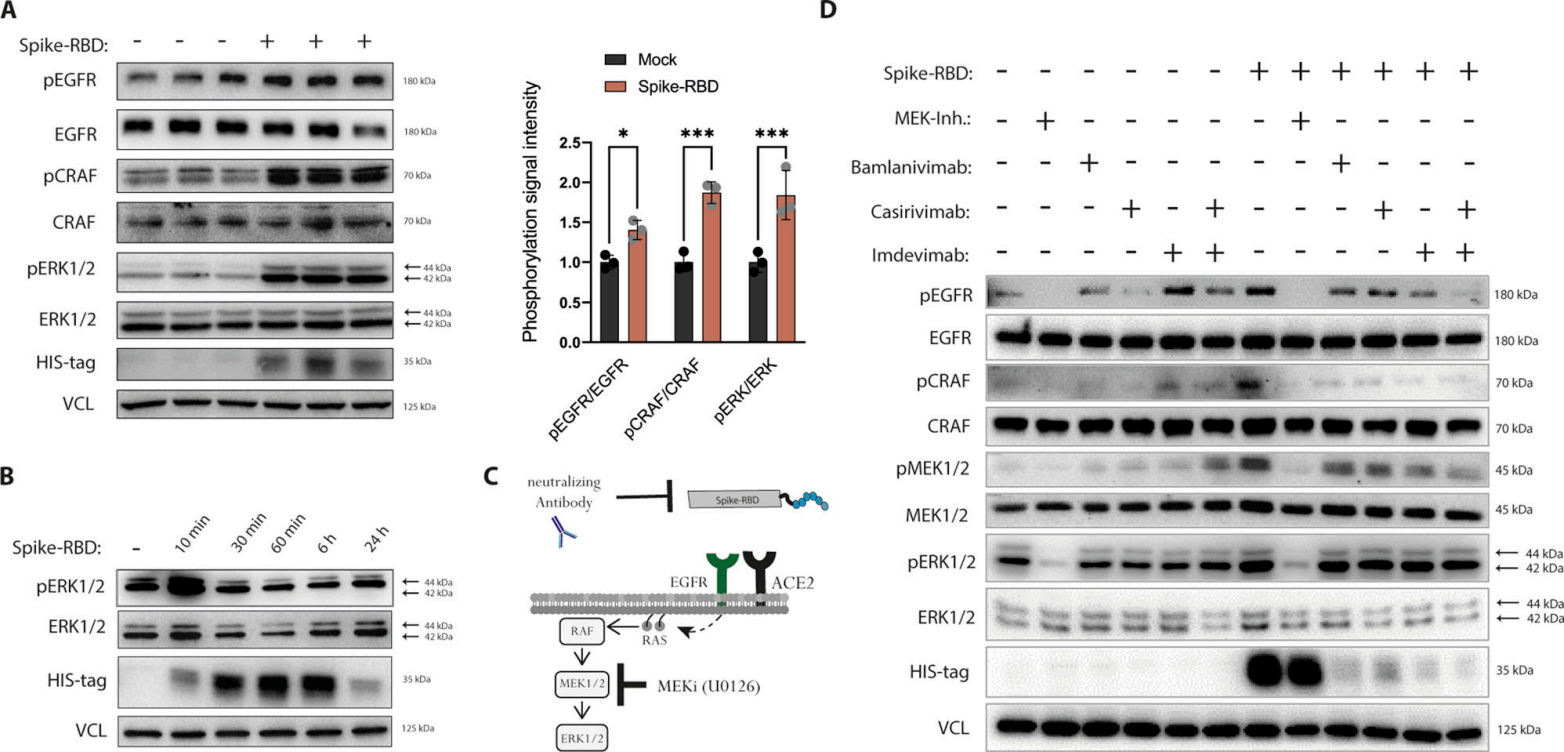
Treatment of Caco-2 cells with the receptor-binding domain (RBD) of the SARS-CoV-2 spike protein (Spike-RBD) leads to epidermal growth factor receptor (EGFR)-MAPK pathway activation. **(A)** Activation of EGFR-MAPK signaling was determined by detecting phosphorylation level of ERK1/2, CRAF, and EGFR by immunoblotting in Caco-2 cells treated with 100 ng Spike-RBD for 10 min. Data for quantification (right graphs) were normalized to total protein amounts of ERK1/2, CRAF or EGFR, and loading controls, respectively. Spike-RBD binding to the cell was proven by immunoblotting using a His-tag specific antibody, whereas vinculin serves as loading control. Asterisks indicate a significant difference from controls (*P* < 0.05, *t* test). Error bars represent the SD of n = 3 independent experiments. **(B)** Caco-2 cells were treated with 100 ng/ml Spike-RBD for the indicated time points and activation of ERK1/2 was detected by immunoblotting using a phospho-ERK1/2-specific antibody. **(C)** Graphical illustration of Spike-RBD-induced EGFR-MAPK activation in the presence of inhibitors and neutralizing antibodies. **(D)** MAPK activation in response to Spike-RBD in the presence of viral entry inhibitors to determine the specificity of Spike-RBD-mediated MAPK activation. Caco-2 cells were treated with Spike-RBD (100 ng/ml) for 10 min in the presence or absence of SARS-CoV-2-neutralising antibodies (bamlanivimab, casirivimab, and imdevimab, all 10 μg/ml added 30 min before Spike-RBD). The MEK inhibitor U0126 (10 μM) was used as a control of MAPK activation. Phosphorylation of EGFR, CRAF, MEK1/2, ERK1/2 was analyzed by immunoblotting using phosphorylation-specific antibodies. Spike-RBD recombinant protein was determined using antibodies raised against His-tag. Source data are available for this figure.

Considering all previous studies on different viruses and the current data on SARS-CoV-2, we aimed at dissecting the role of EGFR–RAS–MAPK axis in viral infection. First, we monitored MAPK activation in response to a treatment with His-tagged recombinant spike receptor binding domain (Spike-RBD) in Caco-2 cells, which have high level of ACE2 (Fig S1A), express *ADAM17*, *TMPRSS2* and *FURIN* (Fig S1B) that were previously described as key players in viral infection, and are highly permissive for SARS-CoV-2 (Zupin et al, 2022) (Fig S1C). Spike-RBD treatment led to an increased activation of the key mediators of MAPK signaling, such as ERK1/2 and CRAF, after 10 min of incubation, whereas at later time points, activation returned to a basal level (Fig 1A and B). Moreover, increased MAPK signaling was also observed in the epithelial lung carcinoma cell line A549 and epithelial pancreas carcinoma cell line MiaPaca2 (Fig S1D). Although both cell lines have reduced level of ACE2 when compared with Caco-2 cells (Fig S1A), thus hinting that other membrane proteins contribute to signal transduction upon binding to the Spike-RBD. As the prototypical upstream activator of MAPK is the EGFR (Fig 1C), we monitored the levels of EGFR activating autophosphorylation occurring at Tyr1068 (Y1068). Immunoblotting data revealed that after 10 min of incubation with Spike-RBD, EGFR activation was increased, correlating with an increased activation of ERK1/2 at the same time point (Fig 1A). To validate that the increase in EGFR-MAPK activation is induced specifically by Spike-RBD, we tested the effects of Spike-RBD in the presence of the neutralizing antibodies bamlanivimab, casirivimab, and imdevimab, all of which target the Spike-RBD sequence and therefore block virion binding to the host cell (Hoffmann et al, 2021). After treating cells with these antibodies and a subsequent incubation with Spike-RBD, immunoblotting data indicated a reduced binding of Spike-RBD to cells and a reduced signaling via the EGFR–CRAF–MEK1/2–ERK1/2 axis, thus confirming the specificity of EGFR-MAPK activation by binding of Spike-RBD (Fig 1D). We also treated the same cells with MEK1/2 inhibitor (MEKi) U0126 and found that the inhibition of MAPK was stronger when compared with neutralizing antibodies (Fig 1D). Interestingly, MEKi not only inhibited the MEK1/2 kinase-mediated activation of ERK1/2, but also had a strong inhibitory effect on the EGFR activation (Fig 1D). These data suggest that an inhibition of MAPK affected a yet unknown feedback loop mechanism regulating MAPK signaling and its membrane-bound upstream activator EGFR.

### Spike-RBD activates MAPK through an ACE2-EGFR cross talk

**Figure 2.**
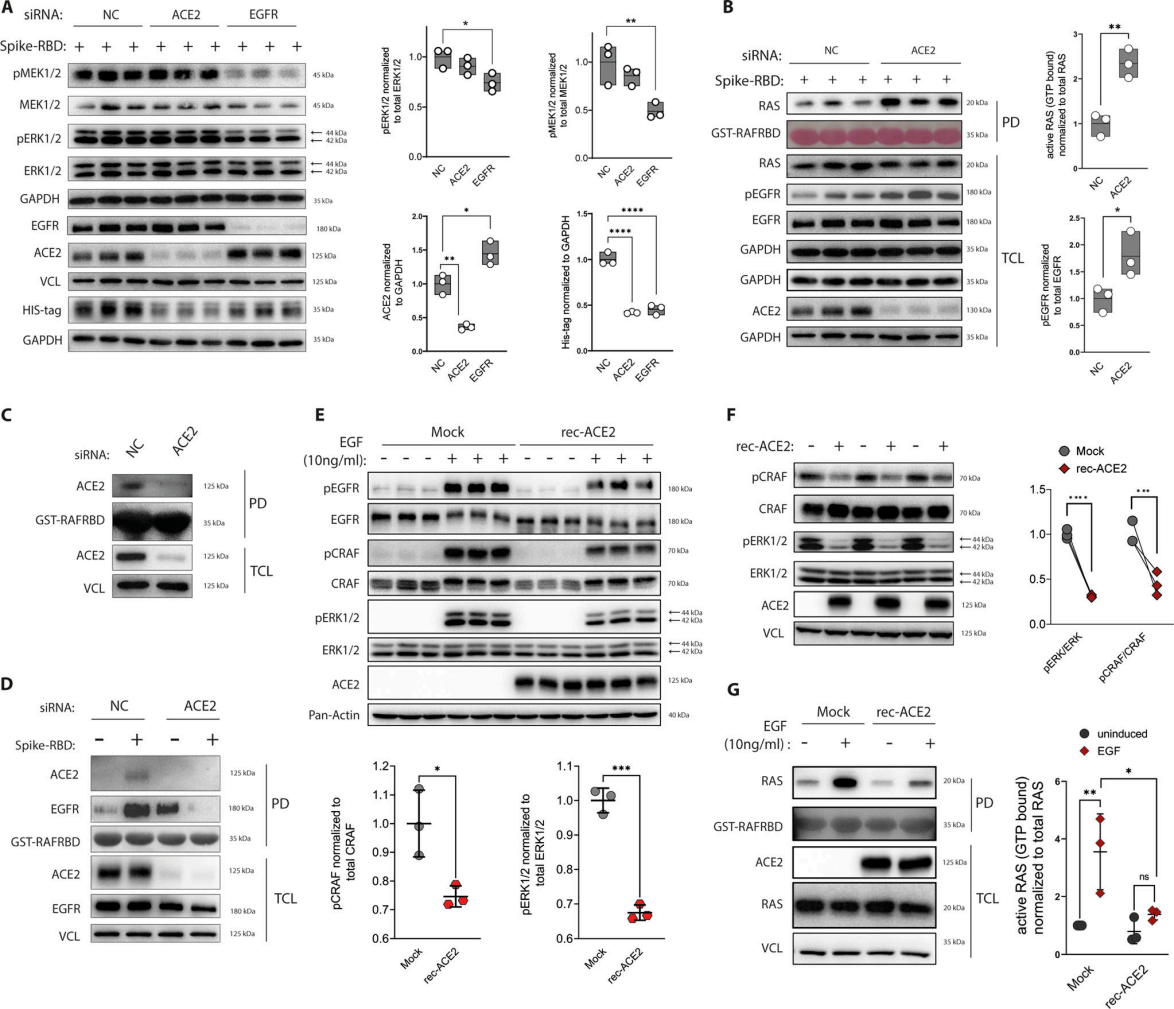
Spike-receptor-binding domain (RBD) binding induces the localization of angiotensin-converting enzyme 2 (ACE2) and epidermal growth factor receptor (EGFR) to active RAS–CRAF kinase complexes. **(A, B, C, D)** The effect of siRNA-mediate knock down (KD) of ACE2 and EGFR on Spike-RBD induced EGFR-MAPK activation in Caco-2 cells. **(A)** Quantification of MAPK activation, ACE2 abundance, and Spike-RBD binding in scramble control (NC), EGFR, and ACE2 KD cells after 10 min of Spike-RBD incubation. Target proteins were detected by immunoblotting using specific antibodies. MEK1/2 and ERK1/2 blots were used as controls for their phospho-forms, whereas GAPDH and vinculin were used as loading controls. **(B)** Quantification of RAS and EGFR activation in ACE2 KD cells after a 10-min Spike-RBD treatment. Active RAS were monitored by pull down assay using as bait GST fused to CRAF kinase RAS-binding domain (CRAF-RBD), followed by immunoblotting. Equal levels of bait levels were verified by Ponceau S staining. Quantification was performed using Image Lab software; phospho-proteins were normalized to their total levels and loading controls and active RAS was normalized to the total RAS levels and loading control. **(C)** Detection of ACE2 in complexes of CRAF kinase was monitored by pull down assay using GST-CRAF-RBD in ACE2 KD and scramble control (NC) Caco-2 cells. ACE2 in pull down samples was tested using ACE2 antibody, whereas vinculin was used as loading control. GST-CRAF-RBD equal loading was performed via Ponceau S staining. **(D)** Detection of ACE2 and EGFR in CRAF kinase complexes in response to ACE2 KD and Spike-RBD stimulation for 10 min. ACE2 and EGFR presence in CRAF complexes were determined by pull down assay using GST-CRAF-RBD. ACE2 and EGFR in pull down samples were monitored using ACE2 and EGFR antibodies, respectively, whereas vinculin was used loading control. GST-CRAF-RBD equal loading was performed via Ponceau S staining. **(E, F, G)** Expression of recombinant ACE2 (rec-ACE2) inhibits RAS-MAPK signaling in HEK293T cells. Activation of the MAPK pathway was detected by immunoblotting using phospho-specific antibodies, EGFR, CRAF, and ERK1/2 blots were used as control for their phospho-forms, whereas pan-actin and vinculin serve as loading controls. **(E)** Activation of MAPK was analyzed in Mock and rec-ACE2 cells either under starvation or EGF induction (10 ng/ml for 10 min). Quantification of CRAF and ERK1/2 activation was done in EGF-induced samples only. **(F)** Activation of MAPK was analyzed in Mock and rec-ACE2 cells only under starvation. **(G)** EGF-induced activation of RAS was monitored by pull down assay using GST-CRAF-RBD, followed by immunoblotting using pan-RAS-specific antibody. Data information: (A, B, E, G), data are represented as single data points and mean ± SD, (F) as single data points, of n = 3 independent experiments. Asterisks indicate a significant difference from controls *P* < 0.05 (*t* test). Source data are available for this figure.

We next aimed to dissect the underlying molecular mechanism of Spike-RBD-mediated EGFR activation. Because both EGFR (membrane receptor and upstream activator of MAPK) and ACE2 (canonical receptor for Spike-RBD) are membrane proteins, we cannot exclude that a cross talk between both membrane receptors affects MAPK activation. To study this hypothesis, we made use of siRNA to knock down (KD) ACE2 and EGFR in Caco-2 cells. Analysis of the MAPK pathway activation in response to gene KD in Caco-2 cells revealed that ACE2 plays a minor role during Spike-RBD-induced activation and suggested that Spike-RBD-induced MAPK activation is at least partly EGFR dependent (Figs 2A and S1E).

Altogether, our data suggest that EGFR may function as a transducer for Spike-RBD-induced activation of MAPK. We further tested the effects of EGFR KD on the binding of Spike-RBD to Caco-2 cells and we identified a reduction in the His-tagged Spike-RBD bound to the host cells (Figs 2A and S1E). Moreover, despite an evident increase in the levels of ACE2 in response to the KD of EGFR, the amount of Spike-RBD bound to host cells was lower than compared with control cells (Figs 2A and S1E). We speculate that EGFR promotes Spike-RBD binding to the cell, either by affecting a conformational state of ACE2 that is competent for Spike-RBD binding, or, considering its high affinity for SARS-CoV-2 Spike protein (Khan & Hatiboglu, 2020; Wang et al, 2021), could interact directly with the Spike-RBD promoting viral attachment to the cell. To complete our analyses of the impact of ACE2-EGFR cross talk on MAPK activation, we also monitored the activation of RAS GTPases, which are molecular switches of the RAF-MAPK pathway (Wennerberg, 2005). Cells treated with Spike-RBD and transfected with ACE2 siRNAs were subjected to pull-down assays using the RAS-binding domain (RBD) of CRAF kinase fused to glutathione S-transferase (GST-CRAF-RBD). As CRAF-RBD binds with highest affinity to active GTP-bound RAS, immunoblotting of pull-down samples showed that RAS activation was enhanced after ACE2 KD. This further indicated that ACE2 KD correlates with increased levels of EGFR activating phosphorylation and activation of downstream RAS GTPase (Fig 2B). Prompted by the identification of a cross talk between EGFR-RAS and ACE2-Spike-RBD, we next monitored the presence of ACE2 and EGFR in active RAS complexes. Using GST-CRAF-RBD as bait, we were able to precipitate ACE2 together with active RAS–CRAF complexes in the absence of Spike-RBD (Fig 2C). Next, we performed a similar pull-down assay in Caco-2 cells stimulated with Spike-RBD and we detected an increased abundance of ACE2 in CRAF complexes (Fig 2D). Moreover, we also detected EGFR in a complex with CRAF in the absence of Spike-RBD stimulation, which increases upon the addition of Spike-RBD. In addition to the use of GST-CRAF-RBD as bait to pull down active RAS and active RAS-containing protein complexes (Caratti et al, 2022), an independent experiment was conducted to validate Spike-RBD-induced complex formation. We transiently transfected Caco-2 cells with a plasmid that contains CRAF fused at its N-terminus to the EGFP and protein lysates were subjected to GFP-TRAP, to monitor proteins that coprecipitate with CRAF recombinant protein. As already showed before, in this set up, the incubation with Spike-RBD led to an increased phosphorylation of both endogenous and recombinant CRAF (rec-CRAF), respectively (Fig S1F). Precipitation of rec-CRAF-EGFP by GFP-TRAP revealed that ACE2 and EGFR were readily found in a complex with rec-CRAF and that Spike-RBD treatment led to an increased presence of ACE2 and EGFR in rec-CRAF complexes (Fig S1G). Also, reduced levels of ACE2 and EGFR were revealed in the unbound fraction (FT) in samples incubated with Spike-RBD when compared with input control (Fig S1G). At this point, although CRAF-RBD precipitates both, we cannot conclude that EGFR and ACE2 may locate in the same RAS–CRAF complex. However, as RAS GTPase activation increases in response to ACE2 KD, it is plausible that ACE2 may act as a negative regulator on EGFR and RAS activation. To assess whether ACE2 functions as a negative regulator of EGFR-RAS activation also under physiological conditions, we used HEK293T cells that are characterized by a low level of endogenous ACE2 (Fig S1A), and we overexpressed recombinant human ACE2 in these cells. Stimulation with EGF led to an attenuated EGFR-MAPK signaling activation in rec-ACE2 HEK293T cells when compared with control cells (Fig 2E), which was also observed under serum-starved conditions (Fig 2F). This further supported our hypothesis that ACE2 may function as negative regulator of MAPK signaling activation, also in the absence of an extracellular stimulus. To see whether ACE2 also impacts active RAS levels, we performed GST-CRAF-RBD PD assays in lysates collected from untransfected and rec-ACE2-transfected HEK293T cells that were serum-starved or induced with EGF. Immunoblotting analyses demonstrated the inhibitory effect of recombinant ACE2 expression on RAS activation, located downstream of EGFR (Figs 2G and S1H).

### Activation of the MAPK promoted by Spike-RBD is reduced by EGFR and MEK1/2 inhibitors

**Figure 3.**
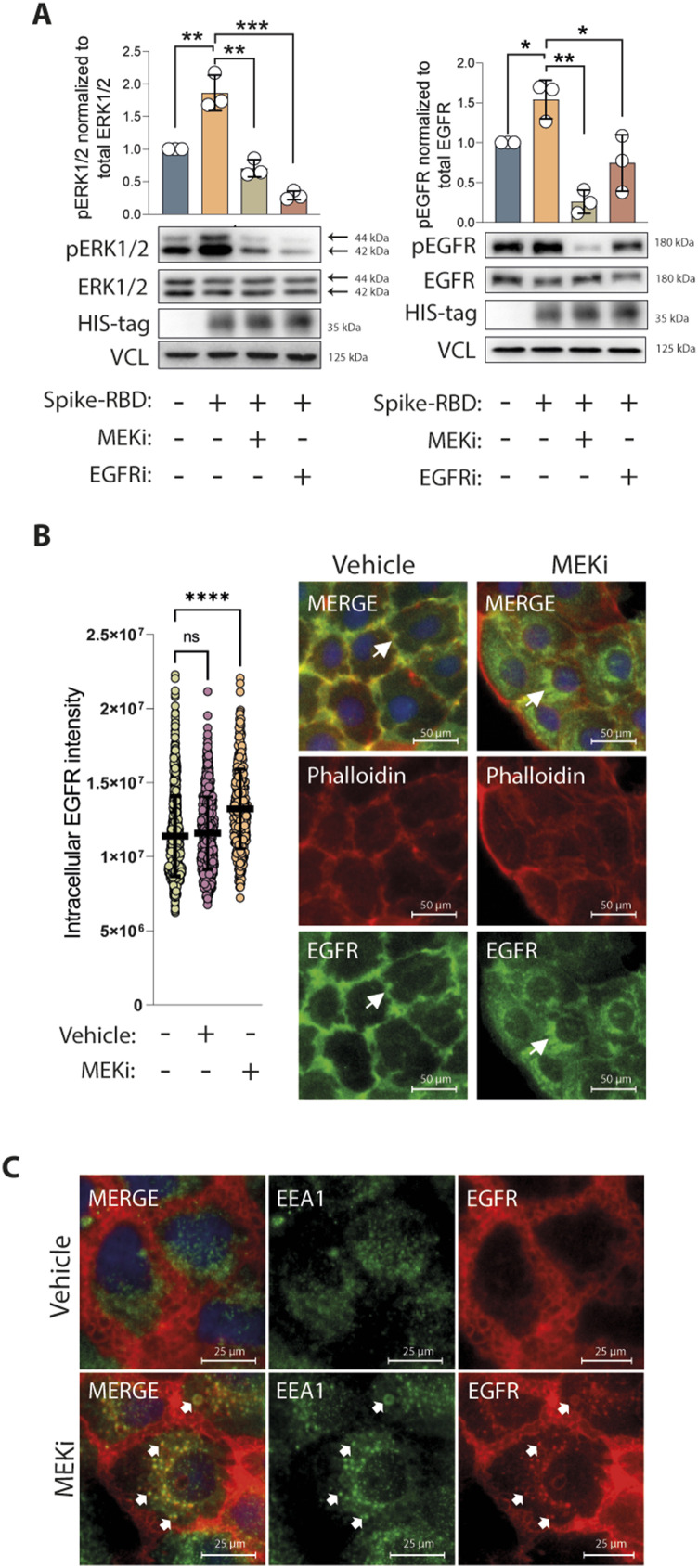
Activation of the MAPK by Spike-receptor-binding domain (RBD) is reduced by epidermal growth factor receptor (EGFR) and MEK1/2 inhibitors. **(A)** Inhibition of ERK1/2 and EGFR activation by the MEK1/2 inhibitor (MEKi) U0126 (10 μM) and EGFR inhibitor erlotinib (5 μM) in the presence of Spike-RBD. Caco-2 cells were treated with the respective inhibitor 30 min before the addition of Spike-RBD (100 ng/ml) for 10 min. Active ERK1/2 and EGFR levels were monitored in total cell lysates by phospho-specific antibodies, whereas total levels were determined with ERK1/2 and EGFR antibodies. Vinculin was used as loading control. Quantification of active levels of ERK1/2 (pERK1/2) and EGFR (pEGFR), normalized to their total levels and loading controls, respectively. Data information: data are represented as single data points and mean ± SD of n = 3 independent experiments. Asterisks indicate a significant difference from controls *P* < 0.05 (*t* test). **(B)** EGFR translocation to vesicular compartments in response to MEKi. Caco-2 cells were treated with MEKi (10 μM) for 24 h and EGFR localization was determined by immunofluorescence using anti-EGFR antibody coupled to AlexaFluor 488 (green). Nuclei are counterstained with DAPI (blue), whereas polymerized actin filaments were stained using phalloidin coupled to AlexaFluor 594 (red). Quantification of intracellular EGFR intensity was performed using the CellProfiler software. Data information: data are represented as single data points per cell and mean ± SD. Asterisks indicate a significant difference from controls *P* < 0.05 (one-way ANOVA). **(C)** EGFR translocation to early endosomes in response to MEKi (10 μM). Caco-2 cells were treated with MEKi for different time points and immunofluorescence staining of EGFR coupled to AlexaFluor 594 (red) and EEA1 coupled to AlexaFluor 488 (green) was performed. Representative picture shows colocalization after 25 min of MEKi treatment. Arrows indicate subcellular localization or colocalization of the tested targets. Source data are available for this figure.

**Figure S2.**
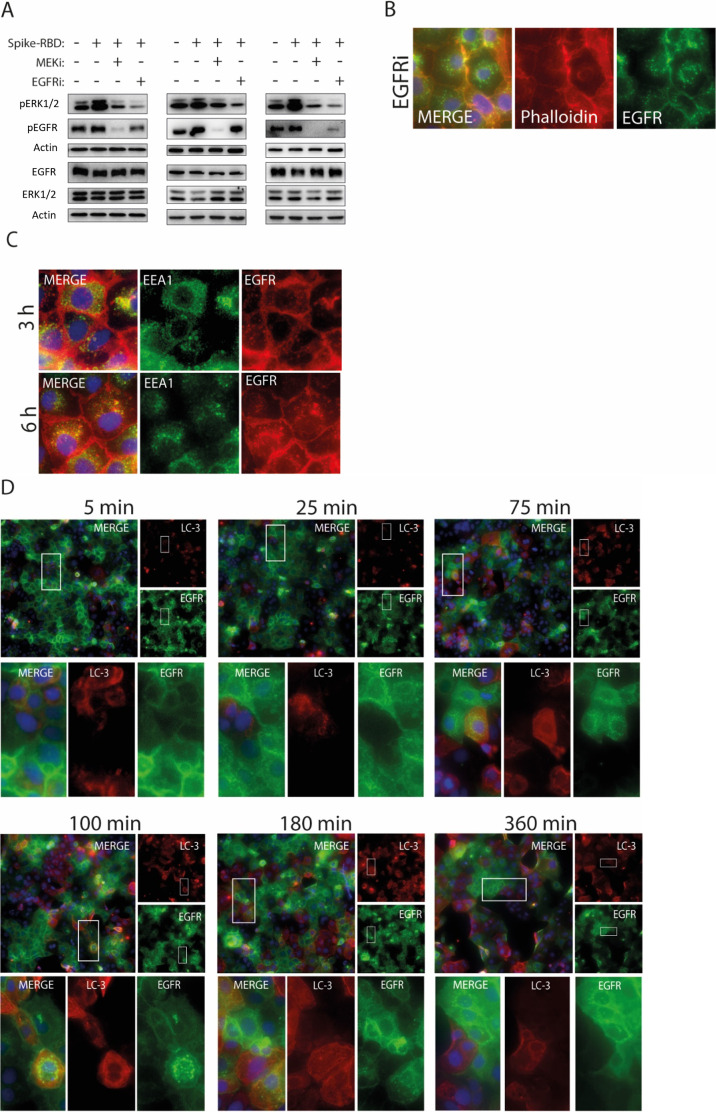
Epidermal growth factor receptor (EGFR) and MEK1/2 inhibitors inhibit MAPK activation and induce EGFR translocation. **(A)** Blots used for quantification of Fig 3A. Inhibition of ERK1/2 and EGFR activation by MEKi (10 µM) and EGFRi (5 µM) in Caco-2 cells treated with 100 ng/ml Spike RBD for 10 min. Active ERK1/2 and EGFR levels were monitored in total cell lysates by phospho-specific antibodies, whereas total levels were determined with ERK1/2 and EGFR antibodies. Vinculin was used as loading control. **(B)** EGFR translocation to vesicular compartments in response to EGFRi (erlotinib). Cells were treated with EGFRi for 24 h and EGFR localization was determined by immunofluorescence using anti-EGFR antibody coupled to AlexaFluor 488 (green). Nuclei are counterstained with DAPI (blue), whereas polymerized actin filaments were stained using Phalloidin coupled to AlexaFluor 594 (red). **(C)** EGFR translocation to early endosomes in response to MEKi. Caco-2 cells were treated with MEKi (10 µM) for 3 or 6 h and immunofluorescence staining of EGFR coupled to AlexaFluor 594 (red) and EEA1 coupled to AlexaFluor 488 (green) was performed. **(D)** Caco-2 cells were treated with MEKi (10 μM) for indicated timepoints and co-staining was performed using EGFR coupled to AlexaFluor 594 (red) and LC-3B coupled to AlexaFluor 488 (green) was performed.

**Figure S3.**
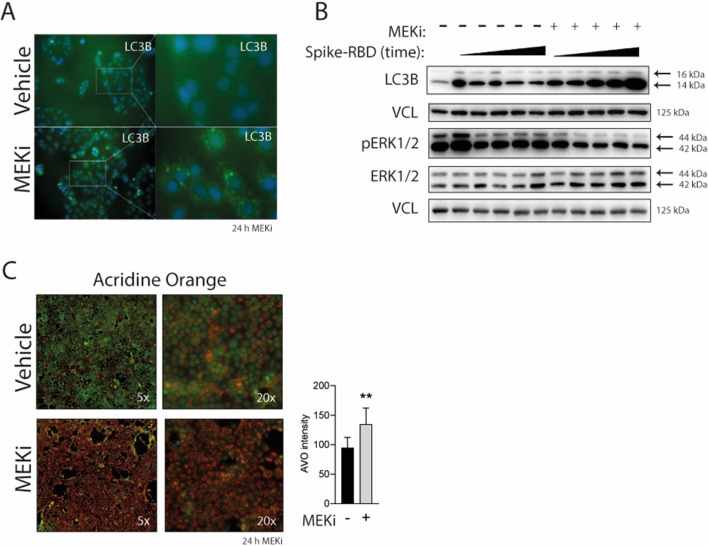
MEK1/2 inhibitor enhances autophagic flux and the amount of acidic vesicles. **(A)** Representative immunofluorescence images of LC3-B coupled to Alexa-488 (green) in control and MEKi (10 μM)-treated Caco-2 cells. Nuclei were counterstained with DAPI. **(B)** Caco-2 cells were treated with Spike RBD (100 ng/ml) in combination with DMSO or MEKi (10 μM) over the period of 24 h. Levels of LC3-B, and ration of LC3-I to LC3-II, were analyzed via immunoblotting at different time points (10, 30, and 60 min, 6 and 24 h). **(C)** Acridine orange was used to label acidic vesicular organelles in control and MEKi-treated cells after 24 h. Acidic vesicular organelle intensity was quantified using image J.

Having shown activation of EGFR-MAPK pathway in response to Spike-RBD, we investigated the effects of pharmacological inhibition of MEK1/2 by using U0126 (MEKi) and of EGFR by using erlotinib (EGFRi) in Caco-2 cells stimulated with Spike-RBD. Both inhibitors reduced activating phosphorylation levels of ERK1/2 and EGFR induced by Spike-RBD, but surprisingly, a stronger reduction of EGFR phosphorylation was detected in response to MEKi when compared with EGFR-specific inhibitor (Figs 3A and S2A). EGFR is located at the plasma membrane, where upon binding EGF, interaction with growth factor receptor-bound protein 2 (GRB2) and activation of RAS-MAPK can be detected. One mechanism affecting EGFR signaling at the plasma membrane is the GRB2-mediated interaction with the E3 ubiquitin-protein ligase casitas B-lineage lymphoma (CBL), leading to EGFR endocytosis and, ultimately, ubiquitination or membrane recycling (Waterman et al, 2002; Jiang et al, 2003). In addition, EGFR translocation is also induced by other factors including different stresses like hypoxia (Shen et al, 2013), oxidative stress (Khan et al, 2006), and treatment with the EGFR inhibitor gefitinib, with the latter leading to endosomal arrest (Blandin et al, 2021) and mitochondrial translocation (Cao et al, 2011) of the EGFR. Taken together with our observation that EGFR activation is reduced in response to MEKi, we were interested in monitoring EGFR localization in response to MEKi treatment. Immunofluorescence data revealed that MEKi promoted EGFR translocation from the membrane into cytoplasmic compartments (Fig 3B), similar to EGFR translocation induced by EGFRi treatment (Fig S2B). We hypothesized that this could lead either to the degradation of EGFR or arresting EGFR in endosomes, blocking its membrane signaling upon activation. To address this question, we performed co-staining of EGFR with the early endosome antigen 1 (EEA1) to monitor endocytosis of EGFR and microtubule-associated proteins 1A/1B light chain 3B (LC3B), a marker for autophagy, at different time points of MEKi treatment. Previous studies showed that several SARS-CoV-2 proteins interfere with the autophagy machinery by reducing autophagic flux, thus allowing SARS-CoV-2 to evade degradation (Koepke et al, 2021; Stukalov et al, 2021). While some studies have reported that MEKi (U0126) inhibits autophagy (Wang et al, 2021) other studies revealed that inhibition of MEK1/2 leads to the activation of the LKB1/AMPK/ULK1 signaling axis and therefore elicits autophagy (Kinsey et al, 2019). Translocation of EGFR into early endosomes was visible as early as after 25 min of MEKi treatment (Fig 3C) and at later time points (3 and 6 h, respectively [Fig S2C]). EGFR did not localize at LC3 positive vesicles at all tested time points (Fig S2D), even though the amount of the LC3B-II autophagy marker was increased during the time of MEKi treatment (Figs S2D and S3A and B). Furthermore, MEKi treatment for 24 h increased the number of acidic vesicle organelles when compared with the control (Fig S3C) Taken together, the treatment with MEKi led to translocation of EGFR into early endosomes, similar to EGFR-specific inhibitors (Blandin et al, 2021). Although an increase of acidic vesicles was observed, we did not observe that EGFR is targeted for degradation by MEKi treatment.

### EGFR is a co-factor for SARS-CoV-2–pseudotyped VSV particles

**Figure S4.**
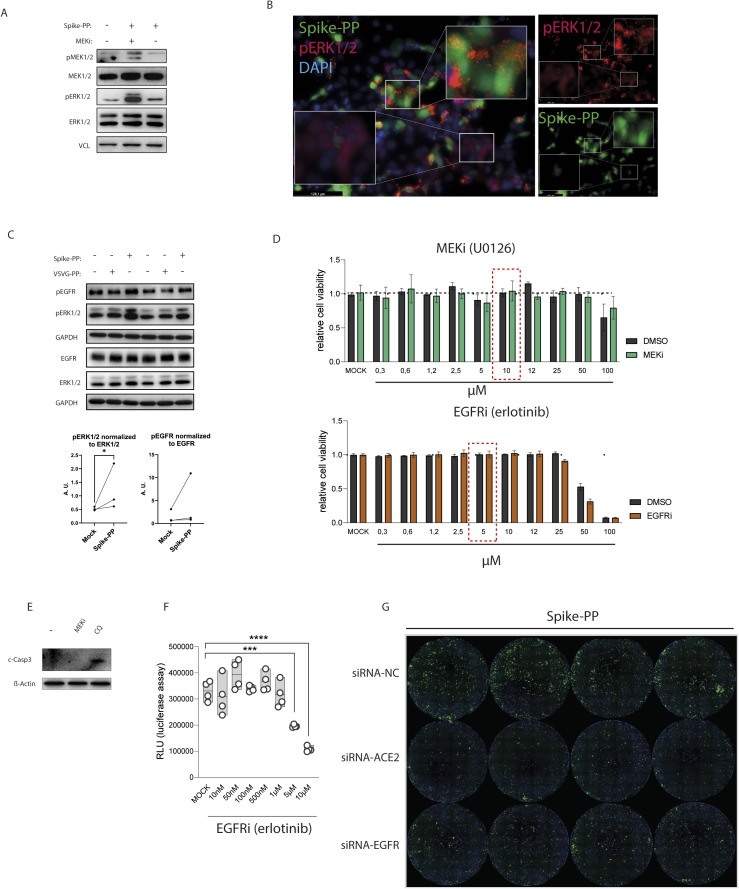
MEK1/2 inhibitor effect on cell viability, and Spike-PP infection in response to epidermal growth factor receptor (EGFR) inhibitor (erlotinib) and angiotensin-converting enzyme 2 (ACE2) and EGFR knockdown (KD), respectively. **(A)** Inhibition of ERK1/2 and MEK1/2 activation by the MEK1/2 inhibitor (MEKi) U0126 (10 μM) in the presence of Spike-PP. Caco-2 cells were treated with MEKi 30 min before the addition of Spike-PP for 10 min. Active ERK1/2 and MEK1/2 levels were monitored in total cell lysates by phospho-specific antibodies, whereas total levels were determined with ERK1/2 and MEK1/2 antibodies. Vinculin was used as loading control. **(B)** Immunofluorescence staining of Caco-2 cells incubated with Spike-PP using phospho-ERK1/2-specific antibody coupled to AlexaFluor 594 (red), whereas infected cells were identified by EGFP expression (green). Nuclei were counterstained using DAPI. **(C)** EGFR and ERK1/2 was monitored using phosphorylation-specific antibodies in protein lysates of Caco-2 cells treated for 10 min with VSV-G-PP and Spike-PP by immunoblotting. EGFR and ERK1/2 blots were used as control for their phospho-forms, whereas GAPDH was used as loading control. Quantification of phosphorylated EGFR and ERK1/2, normalized to total levels and loading control, respectively. **(D, E)** Cytotoxic effects of MEKi (U0126) and EGFRi (erlotinib). **(D)** Relative cell viability of Caco2 cells in the presence of indicated MEKi or EGFRi concentrations as determined by presto blue measurements after 24 h. **(E)** Detection of cleaved caspase 3 (c-Casp3) in total cell lysates from Caco2 cells treated for 24 h with 10 μM MEKi or 100 μM chloroquine (CQ). ß-actin was used as loading control. **(F)** Caco-2 cells were infected with Spike-PP and cell entry was determined by measuring luciferase activity after 24 h in the presence of indicated concentrations of EGFR inhibitor. Asterisks indicate a significant difference from control (**P < 0.01; ***P < 0.001; ****P < 0.0001; one-way ANOVA). **(G)** ACE2 and EGFR KD cells were generated using siRNAs targeting ACE2 or EGFR, respectively. Scramble siRNA was included as negative control (NC). KD and NC cells were infected with Spike-PP and counterstained with Hoechst after 24 h. Replicates were used for quantification of Fig 4B.

**Figure 4.**
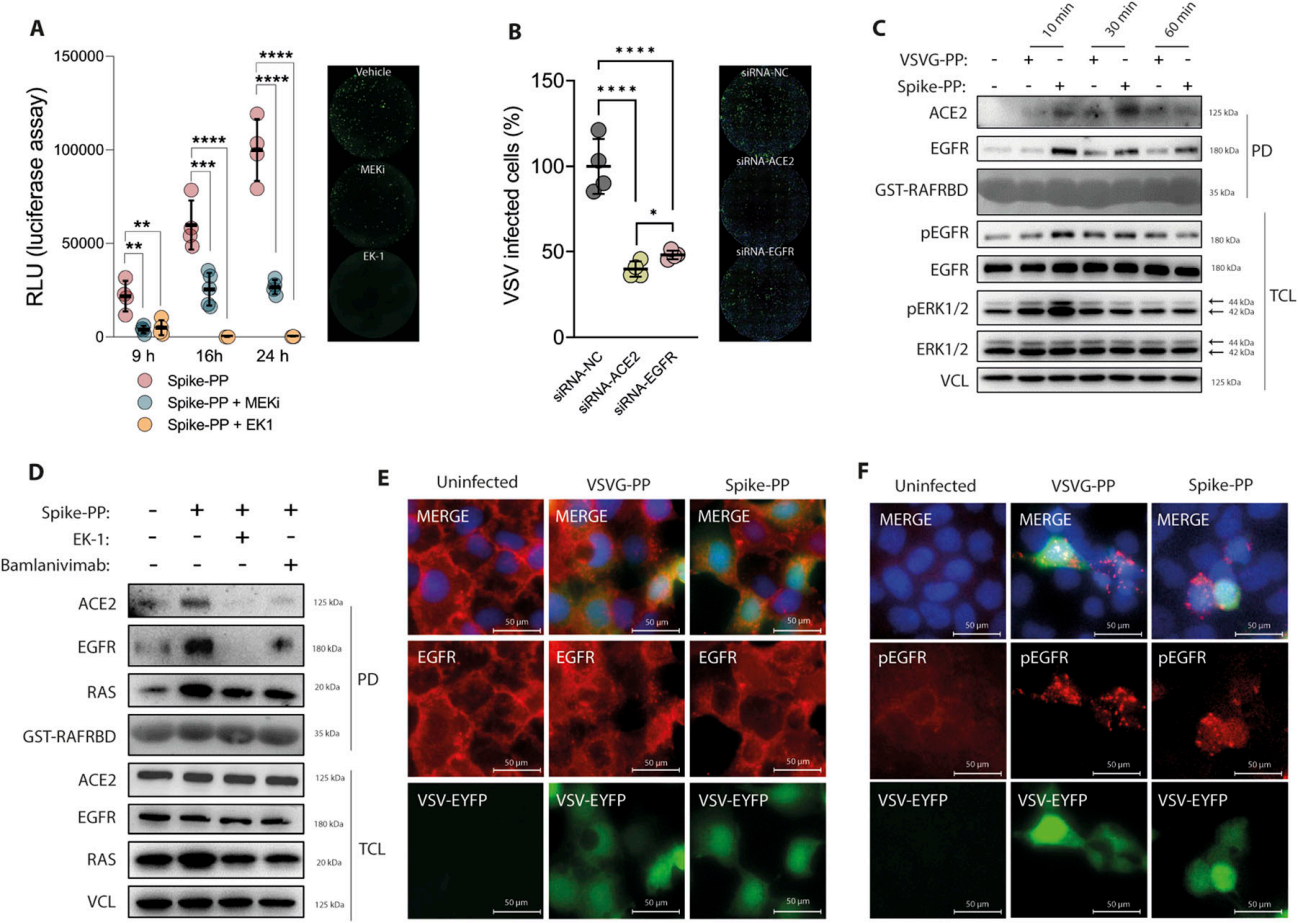
Epidermal growth factor receptor (EGFR) is a co-factor for SARS-CoV-2-pseudotyped VSV particles. **(A)** Inhibition of MEK1/2 reduces SARS-CoV-2 infection in Caco-2 cells. Cells were infected with VSV-based viral particles pseudotyped with SARS-CoV-2-Spike (Spike-PP) and cell entry was determined by measuring luciferase activity after 9, 16, and 24 h in control (vehicle), MEKi (U0126, 10 μM) or EK1 (fusion inhibitor, 5 μM)-treated cells. Representative images on the right showing Spike-PP infected cells (green). **(B)** ACE2 and EGFR knock down cells were generated using siRNAs targeting ACE2 or EGFR, respectively. Scramble siRNA was included as negative control (NC). KD and NC cells were infected with Spike-PP and the percentage of infected cells (green) after 24 h was calculated using Hoechst (blue) to counterstain the nuclei. **(C)** EGFR and ERK1/2 activation was monitored at different time points using phosphorylation-specific antibodies in total cell lysates of control particles pseudotyped with VSV glycoproteins (VSVG-PP) and Spike-PP-treated cells. EGFR and ERK1/2 blots were used as control for their phospho-forms, whereas vinculin was used as loading control. ACE2 and EGFR in CRAF kinase complexes in response to Spike-PP were determined by a pull down (PD) assay using GST-CRAF-RBD followed by immunoblotting, using ACE2 and EGFR antibodies, respectively. Ponceau S staining of GST-CRAF-RBD served as loading control. **(D)** Detection of ACE2 and EGFR in CRAF kinase complexes in response to 10 min of incubation of Spike-PP alone or in combination with EK-1 (5 μM) or bamlanivimab (10 μg/ml). ACE2 and EGFR presences in CRAF complexes were determined by a PD assay using GST-CRAF-RBD followed by immunoblotting, using ACE2 and EGFR antibodies, respectively. Activation of RAS was monitored in the PD samples using a panRAS antibody. Ponceau S staining of GST-CRAF-RBD served as loading control for the bait, and ACE2, EGFR, panRAS, and vinculin served as controls in total cell lysates. **(E, F)** EGFR and phospho-EGFR localization in response to VSVG-PP and Spike-PP incubation for 24 h. Nuclei are counterstained with DAPI (blue), whereas EYFP expression was monitored to identify infected cells. **(E)** EGFR localization was determined by immunofluorescence using an anti-EGFR antibody coupled to AlexaFluor 594 (red). **(F)** Activation of EGFR was monitored by phospho-EGFR Tyr1068-specific antibody coupled to AlexaFluor 594 (red). Data information: (A, B), data are presented as single data points and mean ± SD of n = 4 independent experiments. Asterisks indicate a significant difference from controls *P* < 0.05 (one-way ANOVA). Source data are available for this figure.

Thus far, our data revealed that Spike-RBD modulates EGFR-RAS activation by affecting ACE2-EGFR cross talk. To study this effect in the context of membrane-expressed Spike, we used VSV-based particles pseudotyped with SARS-CoV-2-Spike (Spike-PP) (Capcha et al, 2021). Similar to Spike-RBD, incubation of Caco-2 cells with Spike-PP for 10 min resulted in an increased MAPK signaling (Fig S4A–C), which was abolished by MEKi treatment (Fig S4A). MEKi did not exert cytotoxic effects at our working concentration (Fig S4D and E), therefore possible confounding effects are excluded. To assess whether the inhibition of MAPK may affect viral entry, we first analyzed Spike-PP transduction in either the absence or presence of MEKi. Spike-mediated transduction efficiency was monitored by quantifying GFP expression and luciferase activity at different time points and revealed that MEKi reduced infection rates by 81% after 9 h, 57% after 16 h, and 73% after 24 h (Fig 4A). The pan-coronavirus fusion inhibitor EK-1 (Xia et al, 2019) that blocks Spike rearrangements and entry was used as control and suppressed transduction completely (77% reduction after 9 h, 99% after 16 h and 99% after 24 h). In addition, the EGFR-specific inhibitor erlotinib also reduced the infection of Spike-PP by 40.7% at a concentration of 5 μM and by 67.3% at a 10 μM concentration (Fig S4F). Due to a more potent inhibition on EGFR activation by MEKi when compared with EGFR specific inhibitor (Fig 3A), the EGFR inhibitor provided additional evidence of the importance of EGFR–MAPK axis in a viral infection. In line with a critical role for EGFR in Spike-RBD signaling that we identified, KD of EGFR also led to a significant reduction of Spike-PP infection, shown by a 52.8% reduction of GFP-positive cells when compared with the scramble controls (NC) 24 h postinfection (Figs 4B and S4G). As expected, ACE2 KD strongly reduced the infection of Spike-PP by 62.3% (Figs 4B and S4G).

These results provided unexpected insights into the role of EGFR in SARS-CoV-2 infection and suggest that the virus may hijack EGFR-MAPK signaling for efficient cell entry. To demonstrate that Spike-PPs follow a similar pattern as Spike-RBD in inducing activation of EGFR-MAPK and the formation of a complex that incorporates ACE2, EGFR, and CRAF, we monitored the same targets in cells infected with pseudovirus. Spike-PPs, but not control particles, pseudotyped with VSV-G (VSVG-PPs) led to increased levels of activating phosphorylation of EGFR and consequently of ERK1/2 (Fig 4C). Similar to our observations identified in Spike-RBD experiments, the EGFR-MAPK activation peaked at 10 min after Spike-PP inoculation and was absent in control VSVG-PP-infected cells (Fig 4C). Furthermore, we confirmed that CRAF precipitates ACE2 and EGFR in cells infected with Spike-PP, as analyzed by the GST-CRAF-RBD pulldown assay (Fig 4C). Lastly, the formation of ACE2/EGFR and CRAF putative complex induced by the Spike-PP was inhibited by the addition of the neutralizing antibody bamlanivimab and the pan-coronavirus fusion inhibitor EK-1, the latter demonstrating that Spike-mediated membrane fusion is required to induce the formation of the multi-protein complex (Fig 4D).

An interesting aspect of EGFR-ACE2 cross-talk is that both EGFR (in response to ligand or stress) and ACE2 (e.g., in response to virus attachment) translocate to early endosomes (Inoue et al, 2007; Henriksen et al, 2013). Therefore, we tested whether EGFR may also be translocated from the plasma membrane to other compartments upon exposure to Spike-PP. Immunofluorescence staining of Caco-2 cells revealed that EGFR translocates from the cell membrane into vesicular compartments after exposure to Spike-PP (Fig 4E). However, this effect was not specific for Spike-PPs, as long as it was also observed in cells infected with control VSVG-PPs. Similarly, EGFR activation monitored by phosphorylation of EGFR at Tyr 1,068 by immunofluorescence revealed that activation was similar in cells infected for 24 h with control VSVG-PPs and Spike-PPs (Fig 4F). At this point, we assume that the discrepancy in EGFR activation detected by immunoblotting and immunofluorescence staining is based on time point differences and only the EGFR activation at early time points is specific for SARS-CoV-2 infection.

### Inhibition of EGFR-MAPK signaling reduces infection of authentic SARS-CoV-2

**Figure 5.**
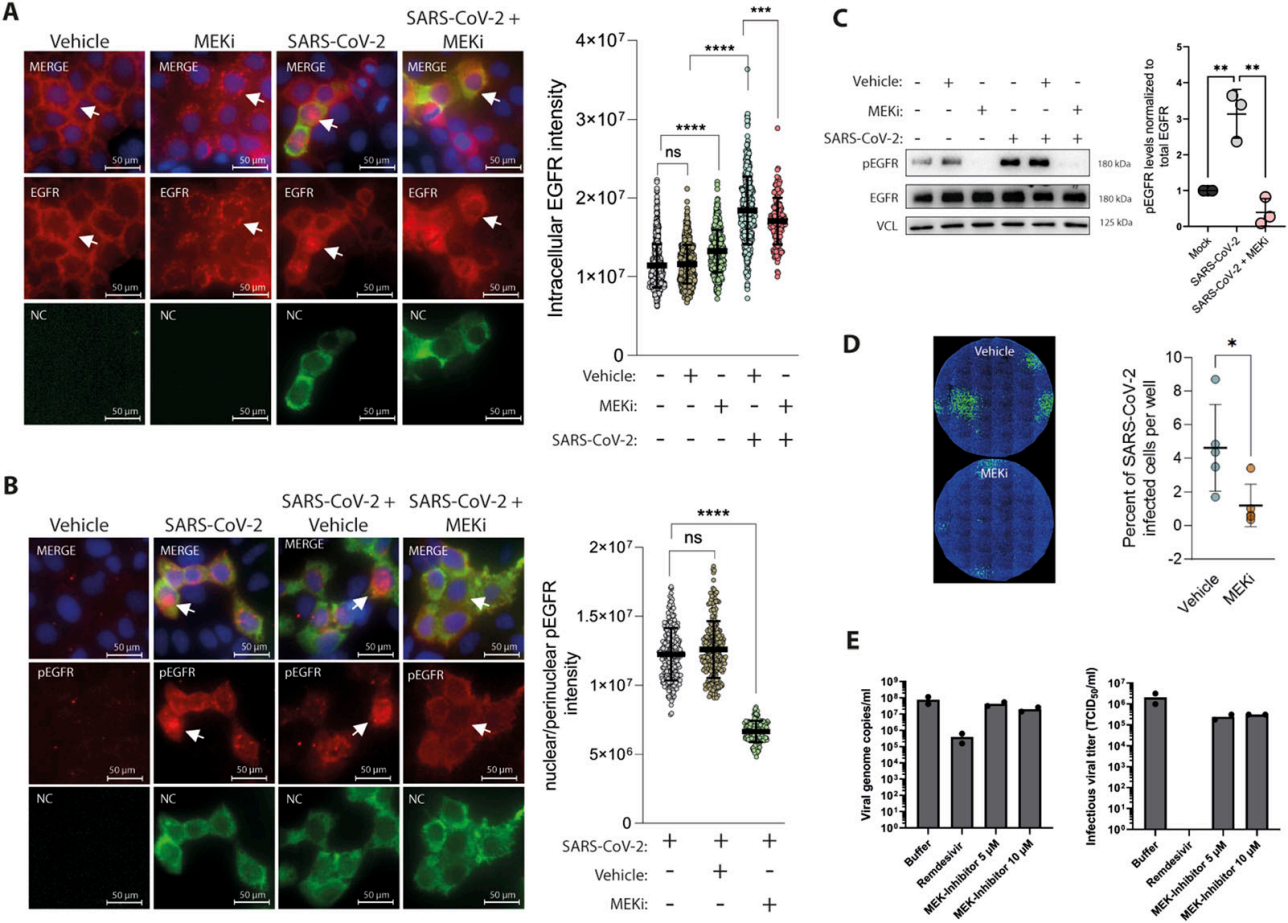
Inhibition of epidermal growth factor receptor (EGFR)-MAPK signaling reduces infection with authentic SARS-CoV-2. **(A, B)** Immunolocalization of EGFR (A) and phospho-EGFR (B), both coupled to AlexaFluor 594 (red) in response to 24 h SARS-CoV-2 infection. Nuclei are counterstained with DAPI (blue), whereas anti-SARS-CoV-2 nucleocapsid (NC) antibody coupled to Alex 488 (green) was used to identify infected cells. Pictures were analyzed using CellProfiler to quantify EGFR translocation and phospho-EGFR intensity within the nuclear/perinuclear space. Data information: data are presented as single data points and mean ± SD of single-cell intensities. Asterisks indicate a significant difference from controls *P* < 0.05 (one-way ANOVA). **(C)** Authentic SARS-CoV-2 leads to increased EGFR activation and is abolished by MEKi. Caco-2 cells were infected with SARS-CoV-2 in the presence or absence of MEKi and were analyzed after 10 min via immunoblotting. Activation of EGFR was detected using phospho-Tyr1068-specific antibody, EGFR blot was used as control for total protein amount, whereas vinculin was used as loading control. Quantification of active phosphorylated EGFR, normalized to total EGFR and loading controls, respectively. Data information: data are presented as single data points and mean ± SD of n = 3 independent experiments. Asterisks indicate a significant difference from controls *P* < 0.05 (*t* test). **(D)** Viral infection was quantified after 24 h in control (vehicle) and MEKi-treated cells. Infected cells were identified by staining for SARS-CoV-2 nucleocapsid protein using anti-NC antibody coupled to Alexa 488 (green) and nuclei were counterstained with DAPI (blue) for quantification using CellProfiler software. Data information: data are presented as single data points and mean ± SD of n = 5 independent experiments. Asterisks indicate a significant difference from controls *P* < 0.05 (*t* test). **(E)** Infection rates in Caco-2 cells were quantified by qRT-PCR (1 d post-infection) and TCID50 (2 d post-infection). Data represents two independent experiments, each performed in triplicates. Arrows indicate subcellular localization of tested targets. Source data are available for this figure.

**Figure S5.**
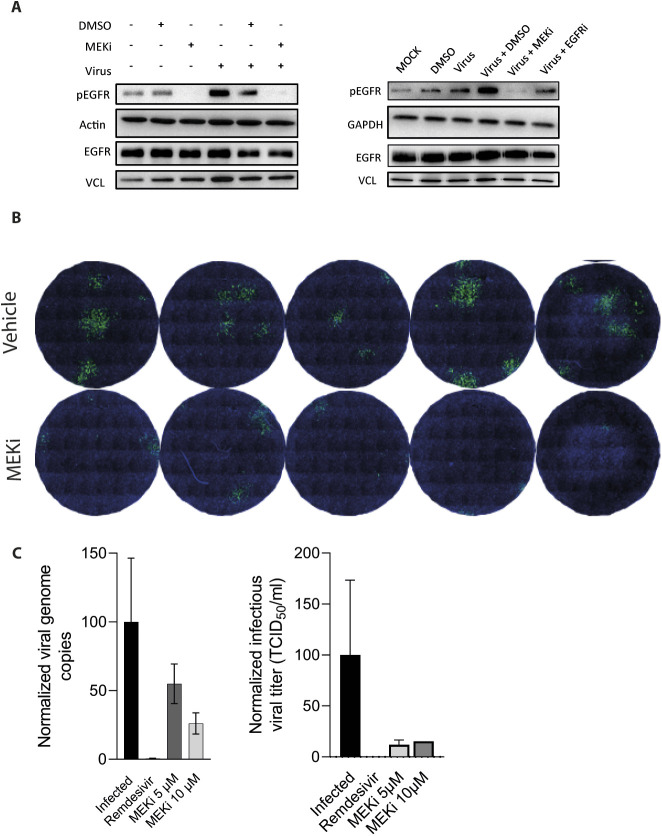
Fluorescence microscopy and immunoblots of Caco2 cells infected with authentic SARS-CoV-2. **(A)** Immunoblots used for the quantification are depicted in Fig 5C. Authentic SARS-CoV-2 induced activation of EGFR after 10 min and inhibition by MEKi. Active EGFR levels were monitored in total cell lysates by phospho-specific antibodies at Tyr1068, whereas total levels were determined with EGFR antibodies. Vinculin, actin, and GAPDH were used as loading control. **(B)** Viral infection was quantified after 24 h in control (vehicle) and MEKi-treated cells. Infected cells were identified by staining for SARS-CoV-2 nucleocapsid protein using anti-NC antibody coupled to Alexa488 (green) and nuclei were counterstained with DAPI (blue). Replicates were used for quantification of Fig 5D. **(C)** Infection rates in Caco-2 cells were quantified by qRT-PCR (1 d postinfection) and TCID50 (2 d postinfection). Data represent two independent experiments, each performed in triplicates. Plots were created with GraphPad Prism 9.

Having observed the impact of Spike-PPs on MAPK activation and EGFR translocation, we performed similar analyses in Caco-2 cells infected with authentic SARS-CoV-2. We first validated the translocation by immunofluorescence at 24 h after infection. As previously shown (Fig 3B), EGFR is predominantly located at the plasma membrane under basal conditions, whereas in response to MEKi, EGFR was internalized (Fig 5A). SARS-CoV-2-infected cells displayed a significant shift of EGFR localization towards cytoplasmic and nuclear compartments, a pattern that was also triggered by the combinatorial treatment of SARS-CoV-2 with MEKi (Fig 5A). However, for the latter, EGFR abundance was reduced when compared with only SARS-CoV-2-infected cells (Fig 5A). Furthermore, SARS-CoV-2-infected cells have increased accumulation of active EGFR (pEGFR) at the nuclear and perinuclear sites which was significantly reduced by the treatment with the MEKi (Fig 5B). Immunoblotting data in lysates collected 10 min after SARS-CoV-2 exposure further demonstrated the inhibitory effect of MEKi on the SARS-CoV-2-induced EGFR activation which may contribute to the change in EGFR localization (Figs 5C and S5A). To further validate EGFR as a cofactor for viral infection and evaluate the EGFR-MAPK pathway as a potential therapeutic target to prevent infection, we analyzed the effect of MEKi treatment on authentic SARS-CoV-2 infection. The percentage of infected cells was determined 24 h after SARS-CoV-2 infection by detecting the viral nucleocapsid (NC) protein via immunofluorescence. Similar to the results with the Spike-PPs, treatment with MEKi reduced the percentage of SARS-CoV-2-positive cells from 4.6% ± 2.3 to 1.2% ± 1.13, respectively (Figs 5D and S5B). This observation was further supported by qRT-PCR-based detection of viral RNA copies in the supernatant of SARS-CoV-2-infected cells. Here, a dosage-dependent reduction of viral copies 1 d after MEK1/2 inhibition (45.05% ± 14.47 for 5 μM and 73.91% ± 7.72 for 10 μM) and a reduced infectious viral titer (TCID_50_) 2 d postinfection (88.1% ± 3.3 for 5 μM and 84.8% for 10 μM) were observed (Figs 5E and S5C). To which extent this is a direct consequence of a reduced viral entry and/or a potential block of viral replication postentry is yet unclear.

## Discussion

Our data revealed that CRAF precipitates ACE2 and EGFR in response to Spike-RBD binding, and thus possibly both may locate and possibly interact in the same protein complex. Our results not only demonstrate a fundamental function of EGFR during SARS-CoV-2 infection, but also revealed a cross talk between EGFR with ACE2, where ACE2 seems to negatively regulate EGFR-RAS GTPase activation. This novel role of EGFR and its association with ACE2 may provide a yet unknown benefit of translocation of SARS-CoV-2 from the plasma membrane via early endosomes. The transient activation of the RAF-MEK1/2-ERK1/2 signaling axis in the early infection phase of SARS-CoV-2 was previous described and the MEK1/2 inhibitor ATR-002 displayed strong anti-SARS-CoV-2 activity (Schreiber et al, 2022). In addition to a more direct implication of EGFR in SARS-CoV-2 infection, some clinical associations further suggested an indirect function of EGFR for lung pathologies in COVID-19. Mutated EGFR is a major driver in lung cancer (Marchetti et al, 2005) and studies showed that cancer patients have an increased risk to develop severe COVID-19 and a threefold increased risk to die by a SARS-CoV-2 infection. However, cancer patients receiving treatment with EGFR inhibitors had lower death rates than cancer patients receiving immunotherapy, chemotherapy or surgery (Albiges et al, 2020; Dai et al, 2020), thus suggesting that EGFR inhibition has a protective effect against severe COVID-19 pathologies. Many survivors of severe COVID-19 display signs of lung fibrosis even month(s) after infection and clinical data identified activation of EGFR as a key regulator inducing fibrosis in lung tissue after SARS-CoV-2 infection (Vagapova et al, 2021), further supporting a critical role of EGFR in COVID-19 and post-acute COVID-19 syndrome (PACS). After the identification of increased EGFR expression in COVID-19 patients, induced by lung injury and STAT1 deficit, first studies now also suggest that the usage of a specific EGFR antibody could reduce inflammation and fibrosis in severe and moderate COVID-19 patients (Londres et al, 2022).

According to previously published data, the cross talk between EGFR and ACE2 identified in our study may be conserved and regulate other biological processes. ACE2 is a key player in the renin–angiotensin–aldosterone system (RAAS), with essential functions in regulating blood pressure, counterbalancing the function of ACE by converting angiotensin (Ang) I to Ang (1–9) and Ang-II into Ang (1–7) (Donoghue et al, 2000; Vickers et al, 2002). The receptor of Ang-II and modulator of the deleterious effects is the G protein-coupled receptor ATR1. In the absence of Ang-II, ATR1 and ACE2 are found in a complex, inhibiting the internalization and subsequent ubiquitination of ACE2 (Deshotels et al, 2014). In the presence of Ang-II and EGF, ATR1 is found in a complex with EGFR (O’Brien et al, 2018) resulting in the transactivation of the latter (Forrester et al, 2016). The involvement of EGFR signaling in RAAS was also demonstrated in several in vivo studies, where its activation was implicated in Ang II-induced hypertrophy (Chan et al, 2015) and pressure overload (Schreier et al, 2016). Therefore, we speculate that a dynamic interaction and complex formation between ATR1, ACE2, and EGFR, depending on ligand or physiological condition, counterbalances their effects.

There are only limited indications in the current literature about an EGFR–ACE2 cross talk. First, in the context of the cardiovascular system, analysis of ACE2 interaction networks in SARS-CoV-2-infected human-induced pluripotent stem cell-derived cardiomyocytes identified EGFR as one of the main altered hub genes (Wicik et al, 2020). Secondly, in the context of cancer progression, a protective function was described for ACE2, again counterbalancing the actions of Ang II, by inhibiting metastasis and tumor growth, in part by inactivating MAPK signaling and epithelial–mesenchymal transition (Ni et al, 2012; Qian et al, 2013). Finally, a potential cross talk between ACE2 and EGFR was also described in non-small-cell lung cancer cells, where mutations in EGFR correlated with an increased expression of ACE2 (Deben et al, 2022). Therefore, the identified cross talk between EGFR and ACE2 could not only pave the route for new strategies to fight viral infections but should also be considered in future cancer and RAAS studies. Among other proteases, ADAM17 was identified to shed ACE2 and promote SARS-CoV-2 infection (Jocher et al, 2022). Interestingly, MAPK activates ADAM17 through ERK1/2 (Zhang et al, 2006), which in turn may induce ACE2 cleavage. In this way, we speculate that ACE2 inhibitory effect on EGFR-MAPK may be abolished, and a subsequent increased activation of EGFR-RAS-MAPK may support viral entry.

In addition to the EGFR-mediated activation of MAPK, we also observed the translocation of the EGFR into the perinuclear space and the nucleus, 24 h post-SARS-CoV-2 infection. The EGFR is a member of the ErbB family of tyrosine kinase receptors with essential functions in epithelial cell physiology by regulating various cellular responses including cell proliferation, survival, growth, and development (Wee & Wang, 2017). Viral-induced activation of EGFR was demonstrated for several viruses including influenza virus (Eierhoff et al, 2010) and Epstein–Barr virus (Kung et al, 2011), besides several others. Furthermore, the turnover of EGFR from the cell surface into early endosomes was reported after infection of several viruses like Zika virus (Sabino et al, 2021) and influenza virus (Eierhoff et al, 2010), serving as signaling mediator and key control point for viral-induced cellular changes for productive or latent infection. In addition, endocytosis of EGFR is well described after ligand binding; here, the ligand-induced activation of EGFR allows the interaction with the E3 ubiquitin-protein ligase CBL, targeting the EGFR for endocytosis by clathrin-mediated endocytosis and clathrin-independent endocytosis. After endocytosis, the fate of EGFR can take different directions, either lysosomal degradation (Duan et al, 2003), translocating back to the plasma membrane, arrest at non-degradative endosomes, translocate to the nucleus or other subcellular locations. On the other hand, EGFR endocytosis can also occur in ligand-independent mechanisms by p38 MAPK-mediated phosphorylation of serine/threonine residues in response to different cellular stresses (Tomas et al, 2017). Interestingly, the endocytotic machinery is also used for SARS-CoV-2 infection. Although the best described way for SARS-CoV-2 cell entry relies on spike activation by TMPRSS2, the virus can also hijack pH-dependent endo/lysosomal host cell proteases such as Cathepsin L to release the viral genome in the cytoplasm; because for the latter, the proteolytic cleavage is happening at a later stage, also ACE2 is found in cytoplasmic compartments after SARS-CoV-2 endocytosis (Inoue et al, 2007; Bayati et al, 2021), a mechanism that could also explain the translocation of EGFR. Therefore, we assume that after SARS-CoV-2 cell entry through the endocytic pathway, EGFR forms a complex with ACE2 and viral particles translocate to early endosomes before viral processing. We speculate that this pathway has a yet unclear benefit for the efficiency of viral infections, because both the KD of EGFR and the pharmacological inhibition and translocation of EGFR from the plasma membrane, by using the MEK1/2 inhibitor U0126, reduced the infectivity SARS-CoV-2.

In this study, we observed that the Spike-RBD of SARS-CoV-2 induces the activation of the CRAF-MEK1/2-ERK1/2 cascade and demonstrated that Spike-RBD-induced MAPK activation occurred in an EGFR-dependent manner in Caco-2 cells. The MAPK pathway regulates a pleiotropy of cellular programs by converting extracellular signals into intracellular responses through the three core kinases: CRAF, MEK1/2, and ERK1/2, respectively. Most viruses exploit cellular signaling pathways for their own replication and propagation, including the MAPK pathway. For example, the JC polyomavirus-induced activation of ERK1/2 is essential for viral transcription, identifying the MAPK-ERK1/2-signaling pathway as a key determinant of JC polyomavirus infection (DuShane et al, 2018). The murine coronavirus mouse hepatitis virus-induced MAPK signaling is essential for viral replication, proven by suppression of viral RNA synthesis by siRNA targeting MEK1/2 and ERK1/2 but also the MEK1/2 inhibitor U0126, which was also used in this study (Cai et al, 2007). The same inhibitor was also used to demonstrate that MAPK signaling is needed for a distinct nuclear export of influenza B virus after infection, thus MEK inhibition impaired viral propagation (Ludwig et al, 2004). Viral-induced MAPK activation is also implicated in inflammation and cytokine production after viral infection, as shown for example for the Japanese encephalitis virus (Raung et al, 2007). Although targeting the EGFR–MAPK axis only partly blocked SARS-CoV-2 infection, it would be interesting to analyze its protective effect in combinatorial treatments with already established therapeutics. Mutations in the SARS-CoV-2 Spike-RBD provide fast viral adaptation to infecting cells and thus targeting Spike-RBD of SARS-CoV-2 to fight the current pandemic may prove to be unreliable in the long term. Therefore, revealing the molecular mechanisms triggered by SARS-CoV-2 binding, necessary for cell entry and production of virions, could provide new avenues for pharmacological treatments of COVID-19 and circumventing virus mutations and adaptations that make vaccines and antibody-based therapies unpredictable when a viral strain adapts.

## Materials and Methods

### Cell culture conditions

Caco-2 cells were cultured in DMEM (Gibco) High Glucose (4.5 mg/l) HEPES and supplemented with 10% FBS (Gibco) and 1% penicillin–streptomycin (Invitrogen) at 37°C in a 5% CO_2_ atmosphere. The following concentrations were used throughout the experiments under serum-starved conditions: 100 ng/ml Spike-RBD (original strain—His-tagged Spike-RBD Protein; InvivoGen), 10 ng/ml EGF (ImmunoTools), 5 μM EK1, 10 μg/ml bamlanivimab (LY-CoV555), 10 μg/ml casirivimab (REGN10933), 10 μg/ml imdevimab (REGN10987), 10 μM MEK1/2-inhibitor (U0126), 5 μM EGFR inhibitor (erlotinib). EK1 was synthesized by the Core Facility Functional Peptidomics, Ulm University Medical Center. Neutralizing antibodies and inhibitors were added 30 min before Spike-RBD/VSV-pseudovirus or SARS-CoV-2 live virus, respectively. If not stated otherwise in the figure legends, Spike-RBD and EGF treatment was always performed for 10 min.

### Transient transfection

pcDNA3.1-hACE2 was a gift from Fang Li (Addgene plasmid # 145033; http://n2t.net/addgene:145033; RRID: Addgene_145033) (Shang et al, 2020a) and CRAF in the pEGFP-c1 backbone was a kind gift from Ignacio Rubio (University Hospital Jena). HEK293T or Caco-2 cells were seeded in 10-cm dishes and transfected with 10 μg of plasmid DNA at a 3:1 ratio, using polyethylenimine transfection reagent. Control cells were only incubated with polyethylenimine (Mock). 24 h posttransfection, the media were changed to serum-starved media for 3 h followed by 10 min incubation with either 100 ng/ml Spike-RBD or 10 ng/ml EGF.

### esiRNA-mediated KD

For esiRNA-mediated KD, Lipofectamine RNAiMAX transfection reagent (#13778; Invitrogen) and esiRNA (1 pmol in 96-well format and 25 pmol for six-well format) were used to transfect cells, following manufacturer’s instructions. Analysis was performed 72 h posttransfection and before harvesting cells were serum-starved for 6 h. esiRNAs targeting human ACE2 (#EHU033081) and EGFR (#EHU076761) were obtained from Sigma-Aldrich, scramble siRNA (D-001206-13-05) was used as negative control and was obtained from Dharmacon (Thermo Fisher Scientific). Sequences are provided in the Supplementary Information section.

### Pseudovirus particle stock production

Pseudoparticle production was performed as previously described (Capcha et al, 2021). Briefly, HEK293T cells were transfected with pCG1_SARS-2-Sdel18 D614G, encoding the Spike protein of Wuhan Hu-1, using Transit LT-1. 24 h posttransfection, the cells were infected with VSVΔG (GFP/luc)*VSV-G at an MOI of 1. The inoculum was removed after 1 h and pseudotyped viral particles were harvested at 16 h postinfection. Cell debris were removed by low-speed centrifugation at 805*g* for 5 min. Residual input particles carrying VSV-G were blocked by adding 10% (Vol/vol) of I1 Hybridoma supernatant (I1, mouse hybridoma supernatant purchased from CRL-2700; ATCC) to the cell culture supernatant.

### Pseudoparticle infection assays

For pseudovirus inhibition experiments, Caco-2 cells were seeded in 96-well plates (10,000 cells/well) 1 d before and were serum-starved for 6 h before incubation with pseudoviruses. MEK1/2 inhibitor U0126 (10 μM) was added 30 min before viral infection. Transduction efficiency was determined after indicated time points by calculating the percentage of EYFP-positive cells using the CellProfiler software. Infected cells were normalized by considering 100% of the experimental replicate with highest transduction efficiency (Broad Institute), and firefly luciferase (FLuc) activity using the luciferase assay. Briefly, Caco-2 cells were lysed with passive lysis buffer (#E1941; Promega) at room temperature and lysates were cleared by centrifugation at 18,400*g* for 4 min. FLuc activity was measured using luciferase assay system (#E1500) from Promega, 10 μl were transferred into a new 96-well plate, and 100 μl of luciferase assay substrate was injected per well and measured using a plate luminometer (Centro LB 960 microplate luminometer; Berthold); enzyme activity was normalized to cell number determined by Hoechst staining.

### SARS-CoV-2 virus stock production

BetaCoV/Netherlands/01/NL/2020 or BetaCoV/France/IDF0372/2020 was obtained from the European Virus Archive. The virus was propagated on Caco-2 cells infected at an MOI of 0.005 in a serum-free medium. In brief, cells were inoculated for 2 h at 37°C and subsequently fresh 10% FCS-containing media was added to the cells. The supernatant was harvested 48 h postinfection upon visible cytopathic effect. To remove debris, the supernatants were centrifuged for 5 min at 1,000*g*, then aliquoted and stored at −80 °C. Infectious virus titer was determined as PFU.

### TCID_50_ endpoint titration

Infectious dose 50 (TCID_50_) of the cell supernatants were determined 24, 48, 72 or 96 h postinfection. Therefore, 20.000 Vero E6 cells were seeded per well in 96-well flat bottom plates in a 100-μl medium (2.5% FCS) and incubated overnight. The next day, 62 μl fresh media containing 2.5% FCS was added to the cells and 18 μl of titrated supernatants were used for infection, resulting in final dilutions of 1:10^1^ to 1:10^12^ on cells in triplicates. The cells were then incubated for 5 d and monitored for cytopathic effect to identify infected wells. TCID_50_/ml was calculated according to the Reed and Muench method.

### qRT–PCR for viral copy number

N (nucleoprotein) RNA levels were determined in supernatants collected from SARS-CoV-2-infected cells 24, 48, 72 or 96 h postinfection. Total RNA was isolated using the Viral RNA Mini Kit (QIAGEN) according to the manufacturer’s instructions. qRT–PCR was performed as previously described (Conzelmann et al, 2020) using TaqMan Fast Virus 1-Step Master Mix (Thermo Fisher Scientific) and an OneStepPlus Real-Time PCR System (96-well format, fast mode). Primers (Chu et al, 2020) were purchased from Biomers and dissolved in RNAse-free water. Synthetic SARS-CoV-2 RNAs (Twist Bioscience) were used as a quantitative standard to obtain viral copy numbers. All reactions were run in duplicates using TaqMan primers/probes.

### Immunoblotting analyses

Caco-2 cells (1 × 10^6^) were seeded in 100-mm Petri dishes, serum-starved overnight, and treated as indicated in the figures. Cells were washed with ice-cold PBS and lysed using a modified RIPA buffer (1% IGEPAL Ca-630, 10% glycerol, 50 mM Tris, 2 mM MgCl_2_, 100 mM NaCl, 20 mM β-Glycerophosphate, 1 mM Na_3_VO_4_, with freshly added EDTA-free Protease Inhibitor Cocktail [Roche]). Subsequently, cell lysates were centrifuged at 18,400*g* for 5 min, and protein concentrations of the supernatants were quantified using the BCA Assay Kit (#23225; Pierce) and adjusted to the sample with the lowest concentration to achieve equal loading. Proteins were separated by SDS–PAGE in 10% PAA gels under reducing conditions and transferred on to a nitrocellulose membrane (#1620112; Bio-Rad). Membranes were blocked with 5% BSA in TBST for 1 h at room temperature followed by incubation with primary antibodies overnight at 4°C and 1 h at room temperature with secondary antibodies. The following antibodies and dilutions were used: anti-ERK1/2 (#9102, 1:1,000; Cell Signaling), anti-pERK1/2 Thr202/Tyr204 (#4370, 1:1,000; Cell Signaling), anti-His-tag (#66005, 1:1,000; Proteintech), anti-EGFR (#4267, 1:1,000; Cell Signaling), anti-pEGFR Tyr1068 (#3777, 1:1,000; Cell Signaling), anti-CRAF (#9422, 1:1,000; Cell Signaling), anti-pCRAF Ser338 (#9427, 1:1,000; Cell Signaling), anti-MEK1/2 (#9122, 1:500; Cell Signaling), anti-pMEK1/2 Ser217/221 (#9154, 1:500; Cell Signaling), anti-ACE2 (#21115-1-AP, 1:1,000; Proteintech), anti-LC3B (#3868, 1:1,000; Cell Signaling), anti-GFP (#2956, 1:1,000; Cell Signaling), anti-RAS (#2668896, 1:1,000; Millipore) anti-vinculin (#sc-73614, 1:2,000; Santa Cruz), anti-GAPDH (#60004, 1:2,000; Proteintech) and anti-ß-actin (#A2228, 1:1,000; Sigma-Aldrich). Secondary antibodies were horseradish peroxidase-conjugated goat anti-mouse IgG (#P0447; Dako) and horseradish peroxidase-conjugated goat anti-rabbit IgG (#A6154; Invitrogen). Signal detection was performed using Immobilon Forte Western HRP substrate (#WBLUF0500; Millipore) and recorded with ChemiDoc MP Imaging System (Bio-Rad).

### Active RAS pulldown (PD) assay

RAS pulldown assay was performed as previously described (Caratti et al, 2022). Briefly, Caco-2 cells (1 × 10^6^) were seeded in 100-mm Petri dishes, serum-starved overnight with subsequent treatment as indicated in the figures. For PDs in KD samples 0.2 × 10^6^ cells were grown in six-well plates and lysates of two wells were combined. Before lysis, cells were washed twice with ice-cold PBS, lysed with modified RIPA buffer, and cleared at 18,400*g* for 5 min at 4°C. The total cell lysates (TCL) were normalized by the BCA assay. Non-denatured cell lysates were further subjected to PD, using GST fused to the RAS-binding domain of the CRAF kinase (GST-CRAF-RBD) as bait. PD and TCL samples were mixed with Laemmli buffer and denatured at 95°C for 5 min. Immunoblotting analyses were performed using the antibodies indicated in figure and presented above.

### Live-cell imaging

Live-cell imaging was performed in Caco-2 cells that had been plated in a 96 well and treated for 24 h with MEK1/2 inhibitor (U0126; 10 μM) or DMSO. 5 μg/ml acridine orange was added to the cells for 30 min. Cells were washed once with prewarmed PBS and pictures were acquired using a Leica DMI6000B microscope.

### IP of GFP-fusion proteins (GFP-TRAP)

Caco-2 cells overexpressing recombinant EGFP-CRAF were used to precipitate CRAF-binding partners under starved and Spike-RBD-pulsed conditions using GFP-TRAP agarose beads (ChromoTek), according to the manufacturer´s instructions. Briefly, 25 μl of GFP-TRAP agarose beads were incubated with TCL containing 150 μg of protein for 1 h at 4°C, with constant mixing. Beads were sedimented by centrifugation at 2,500*g* for 5 min at 4°C, the supernatant was removed and saved for further analysis as flow-through (FT) fraction. The beads were washed twice with modified RIPA buffer by centrifugation at 2,500*g* for 5 min at 4°C. Sedimented beads were resuspended in 50 μl 2x Laemmli buffer and denatured for 5 min at 95°C. Immunoprecipitated proteins, input proteins, and flow-through fraction were analyzed by immunoblotting with specific antibodies.

### Immunofluorescence staining

For all IF experiments Caco-2 cells were seeded (10,000/well) in a 96-well plate 1 d before, serum-starved for 6 h, treated with MEKi (10 μM) alone or in combination with either Spike-PP or authentic SARS-CoV-2. For the combinatorial treatment of Spike-PP and SARS-CoV-2 with MEKi, MEKi was added 30 min before. After 24 h, cells were analyzed by immunofluorescence. Briefly, the cells were fixed in 4% PFA, permeabilized using 0.2% Triton-X100 and incubated with a blocking solution (2% BSA, 5% goat serum, 0.4% Triton-X100 in PBS) for 1 h at room temperature. The cells were incubated overnight at 4°C with anti-EEA1 (#68065, 1:250; Proteintech) anti-EGFR (#4267, 1:700; Cell Signaling), anti-pEGFR Tyr1068 (#3777, 1:500; Cell Signaling), anti-LC3B (#3868, 1:500; Cell Signaling), anti-LC3B (#sc-398822, 1:50; Santa Cruz), anti-SARS-CoV-2 Nucleocapsid (#40143-MM05, 1:1,000; SinoBiological) diluted in a blocking solution. After extensive washing, the cells were incubated with secondary antibodies (1:1,000 diluted) coupled to fluorophores: anti-rabbit AlexaFluor 488 (#4412; Cell Signaling), anti-rabbit AlexaFluor 594 (#A-11037; Invitrogen) and anti-mouse AlexaFluor 488 (#A-11001; Invitrogen). Nuclei were counterstained with DAPI (#10184322; Invitrogen), and when indicated in the figure actin filaments were counterstained using phalloidin-AlexaFluor 546 (#A22283; Thermo Fisher Scientific). Cells were washed three times before scanning the plates using the ImageXpress Confocal High-Content Imaging System (Molecular Devices) and analysis of the acquired picture series using the CellProfiler software (Broad Institute).

### Statistical analysis

All results were presented as means ± SD from three independent experiments and performed using GraphPad Prism software version 8.0, *P*-values for all data were determined using *t* test and one-way ANOVA. *P*-values are shown as * = *P* < 0.05, ** = *P* < 0.01, ***P = 0.001, ns, not significant.

## Data Availability

All data are available from the corresponding author on reasonable request.

## Supplementary Material

Reviewer comments
