# Supplementary figures and images for: ACE2-EGFR-MAPK signaling contributes to SARS-CoV-2 infection
Source: Life Sci Alliance. 2023 Jul 4;6(9):e202201880. doi: 10.26508/lsa.202201880 (PMC10320016; doi:10.26508/lsa.202201880)

**Fig 1A**

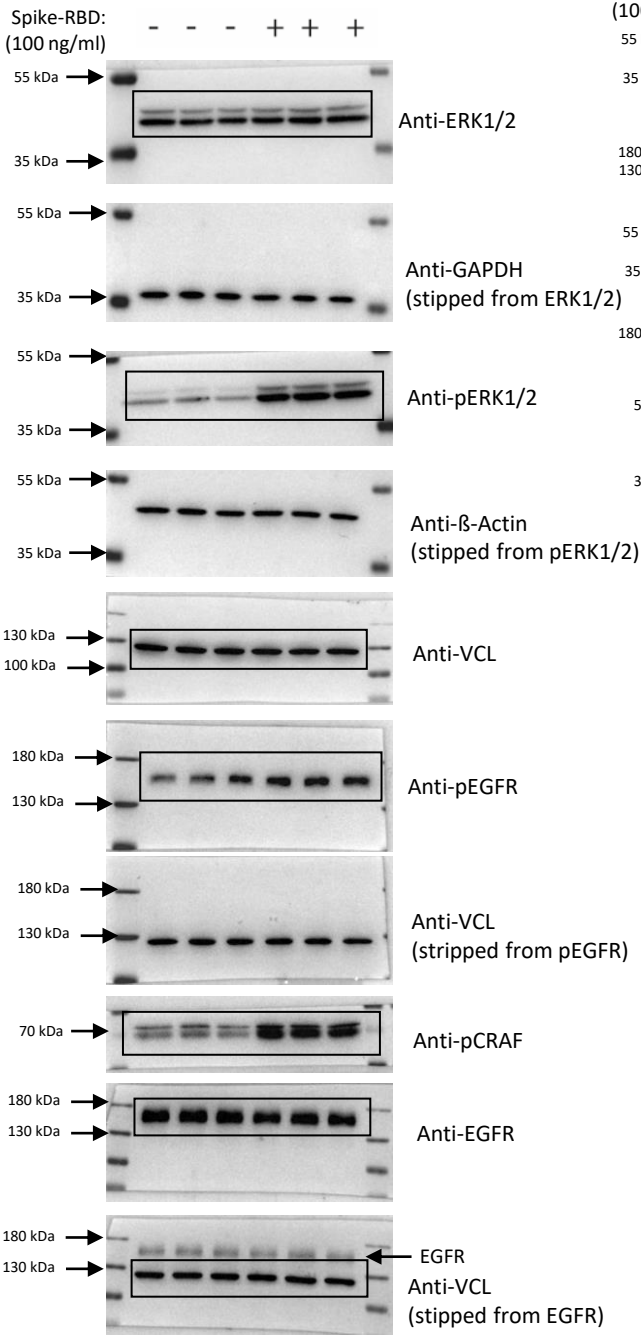

**Fig 1B**

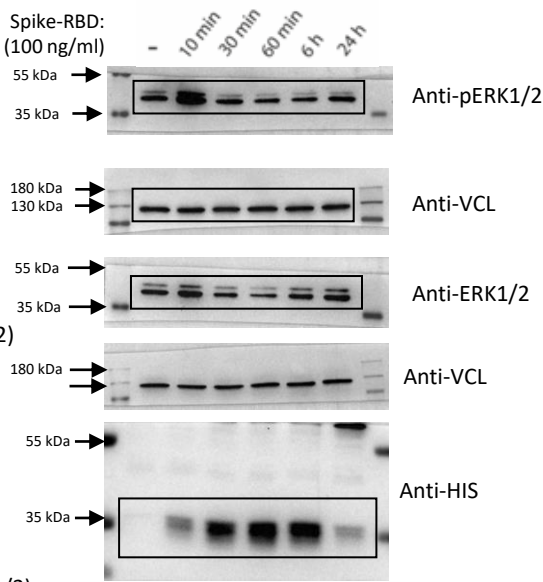

Fig 1D

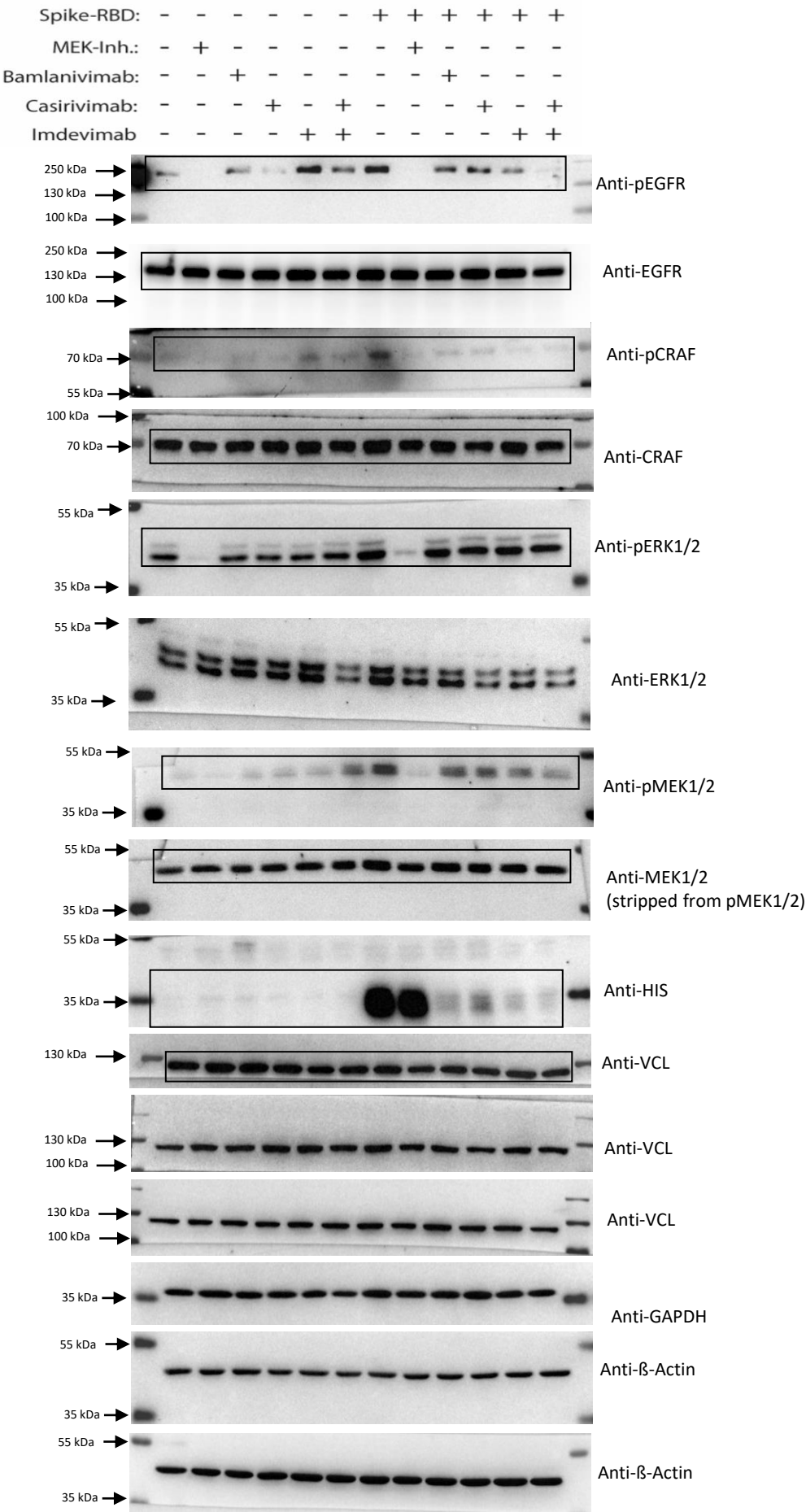

Supplement: Supplementary file 1 [file LSA-2022-01880_SdataF1.pdf]

Fig 2A

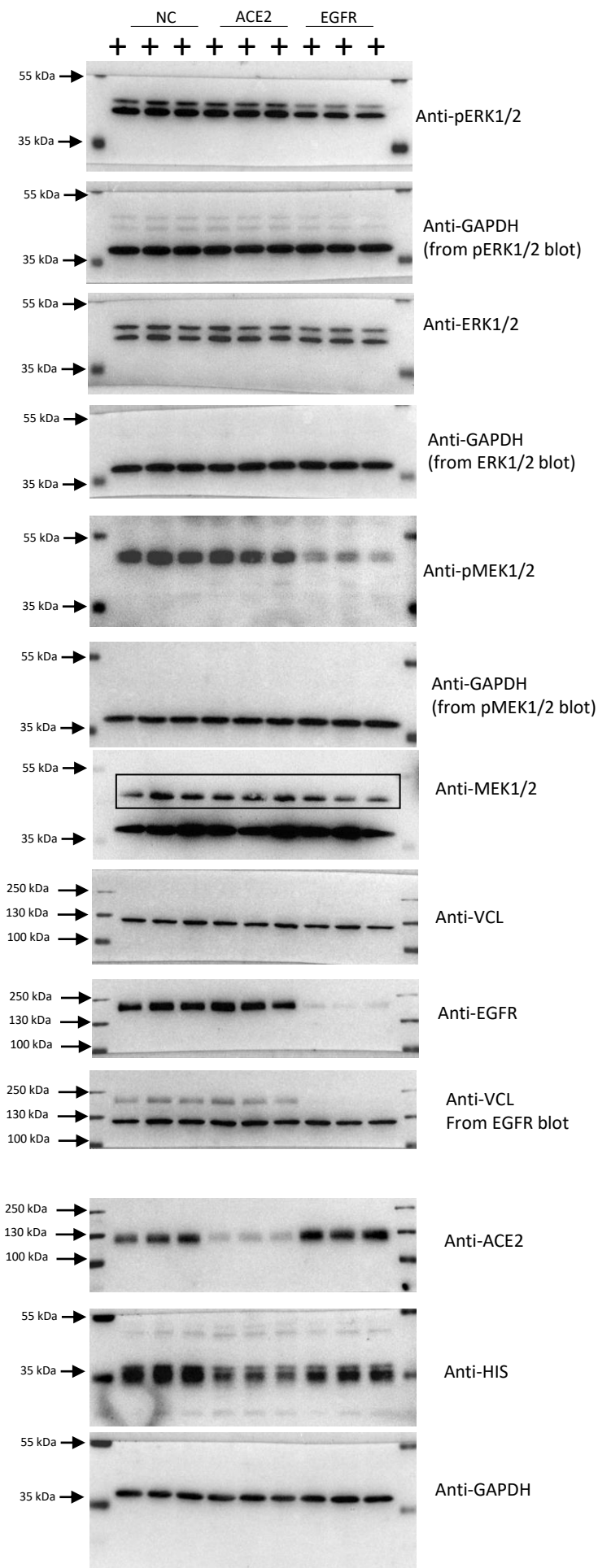

Fig 2B

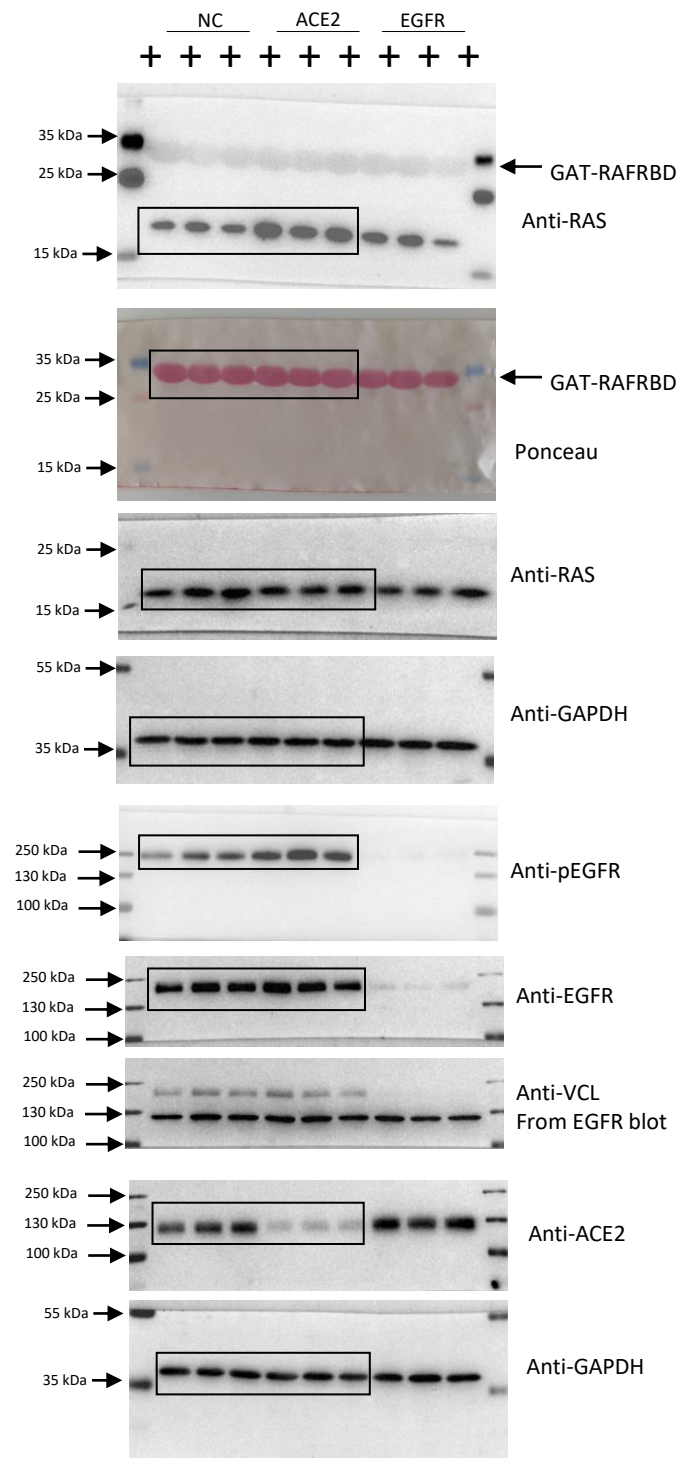

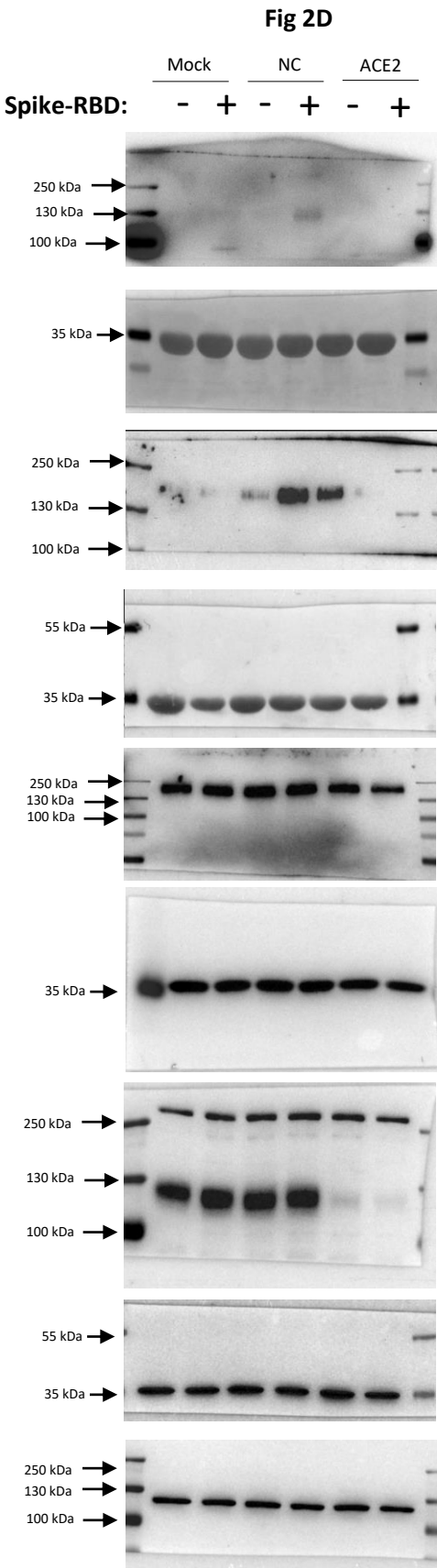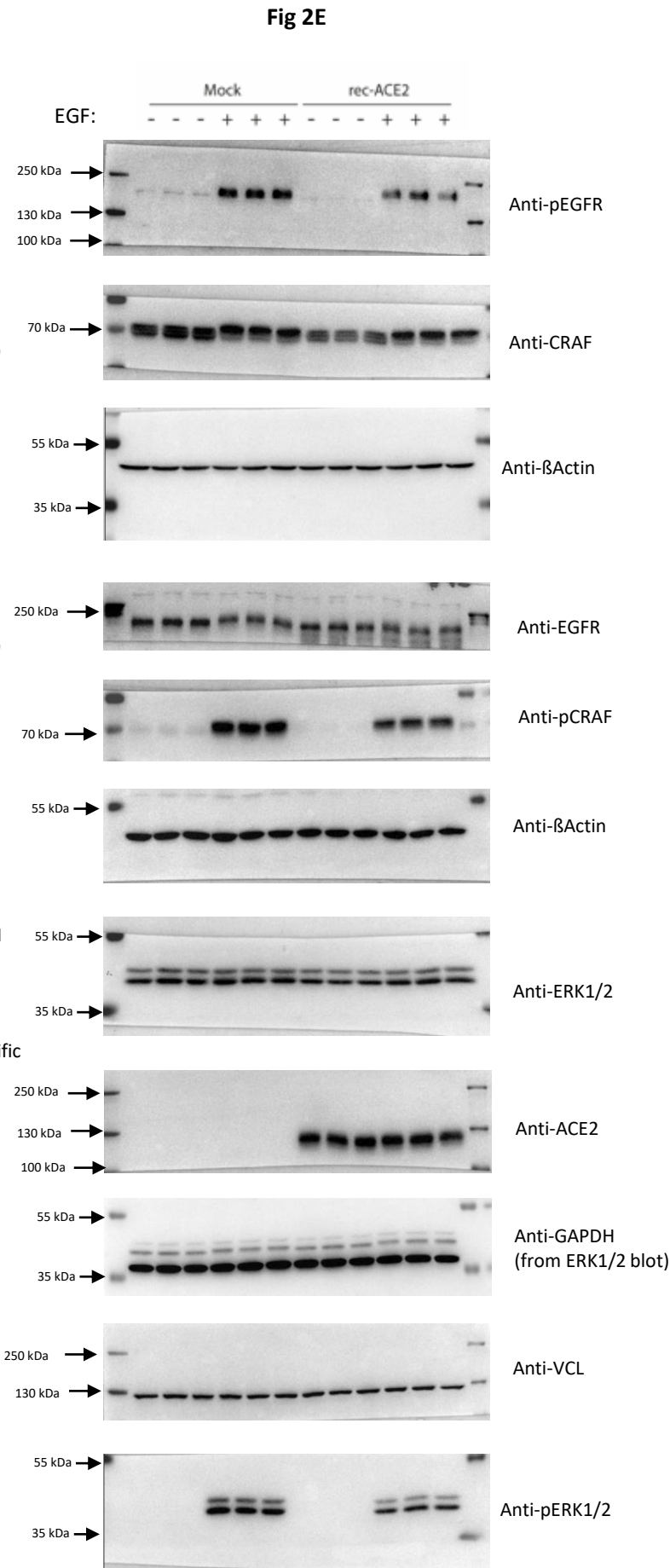

Fig 2F

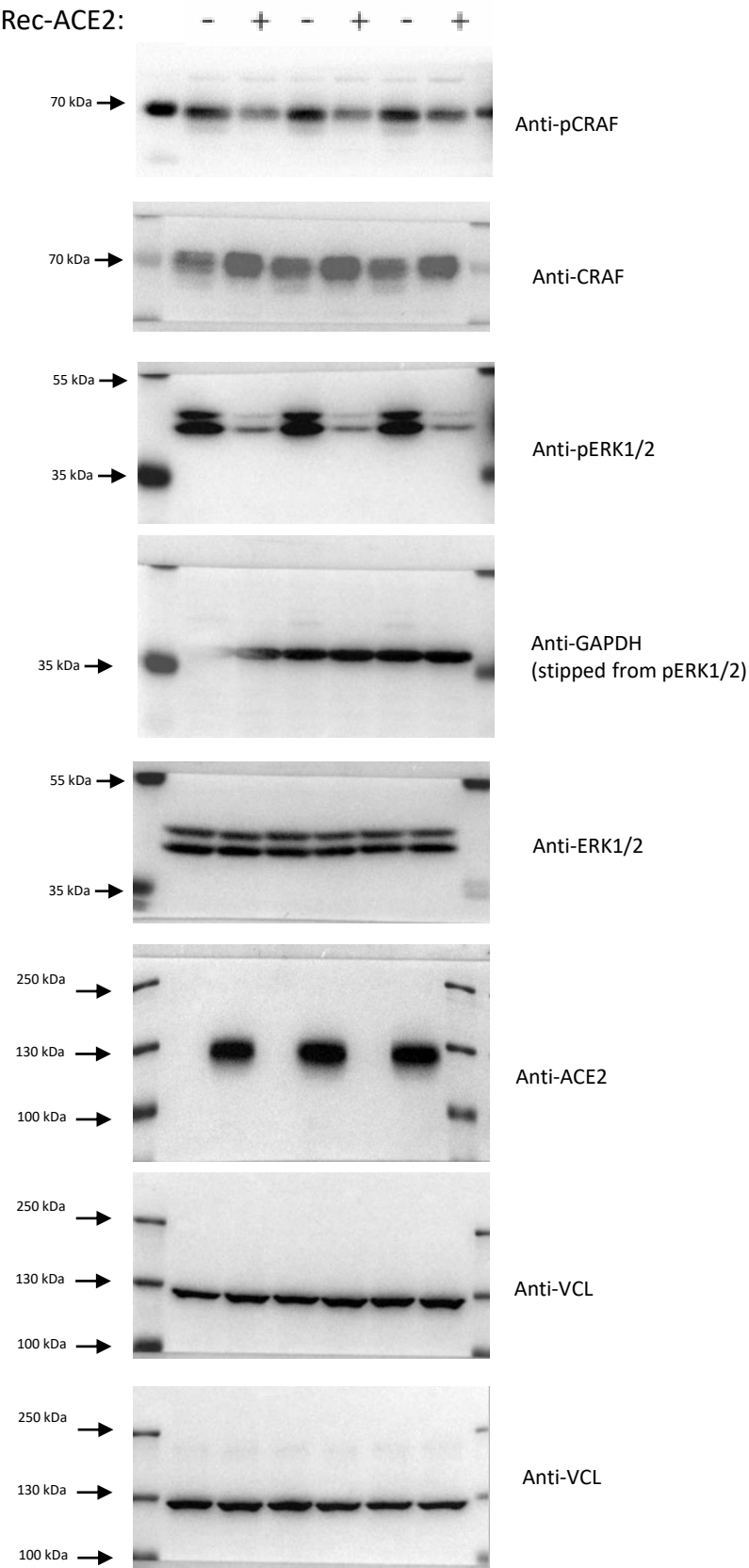

Fig 2G

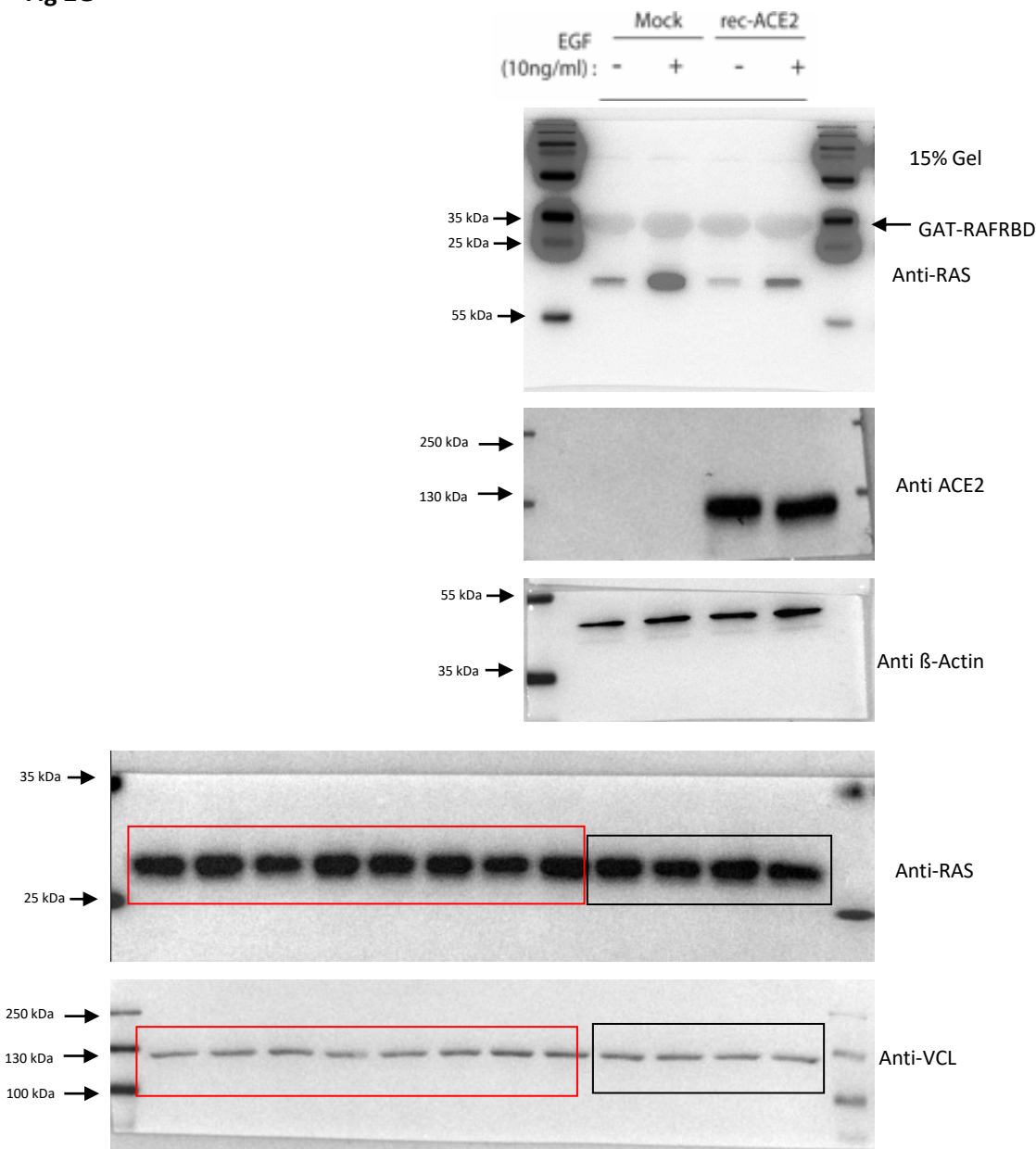

N1 and N2 presented in Supplementary Fig. S1H

Supplement: Supplementary file 2 [file LSA-2022-01880_SdataF2.pdf]

Fig 3A

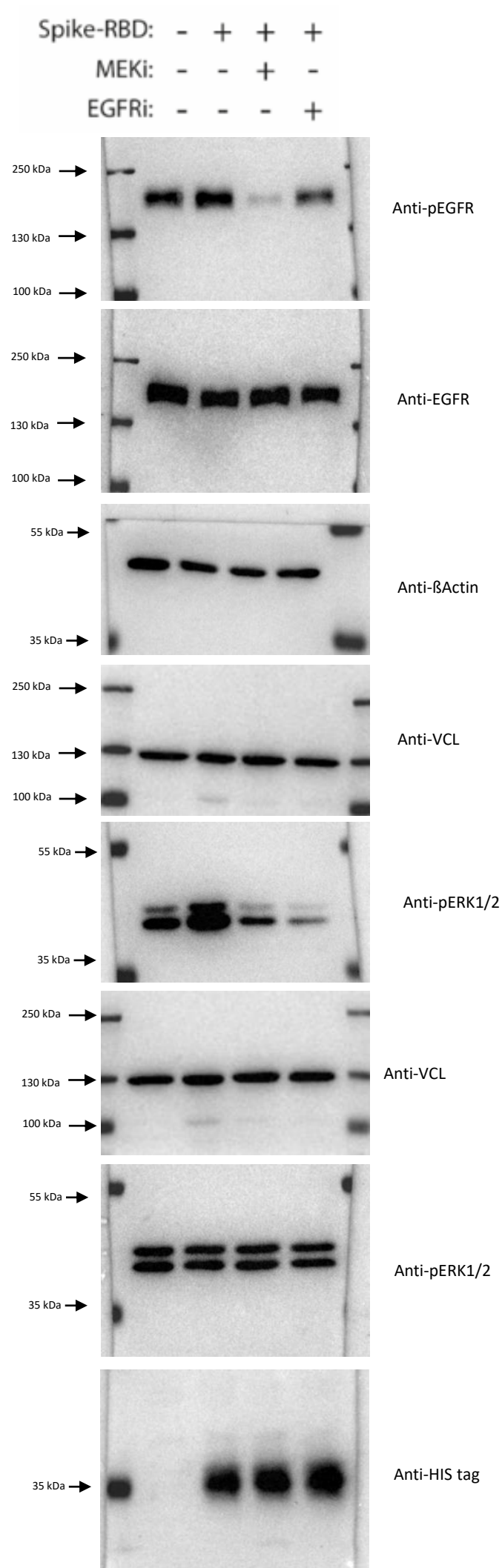

Fig 3B

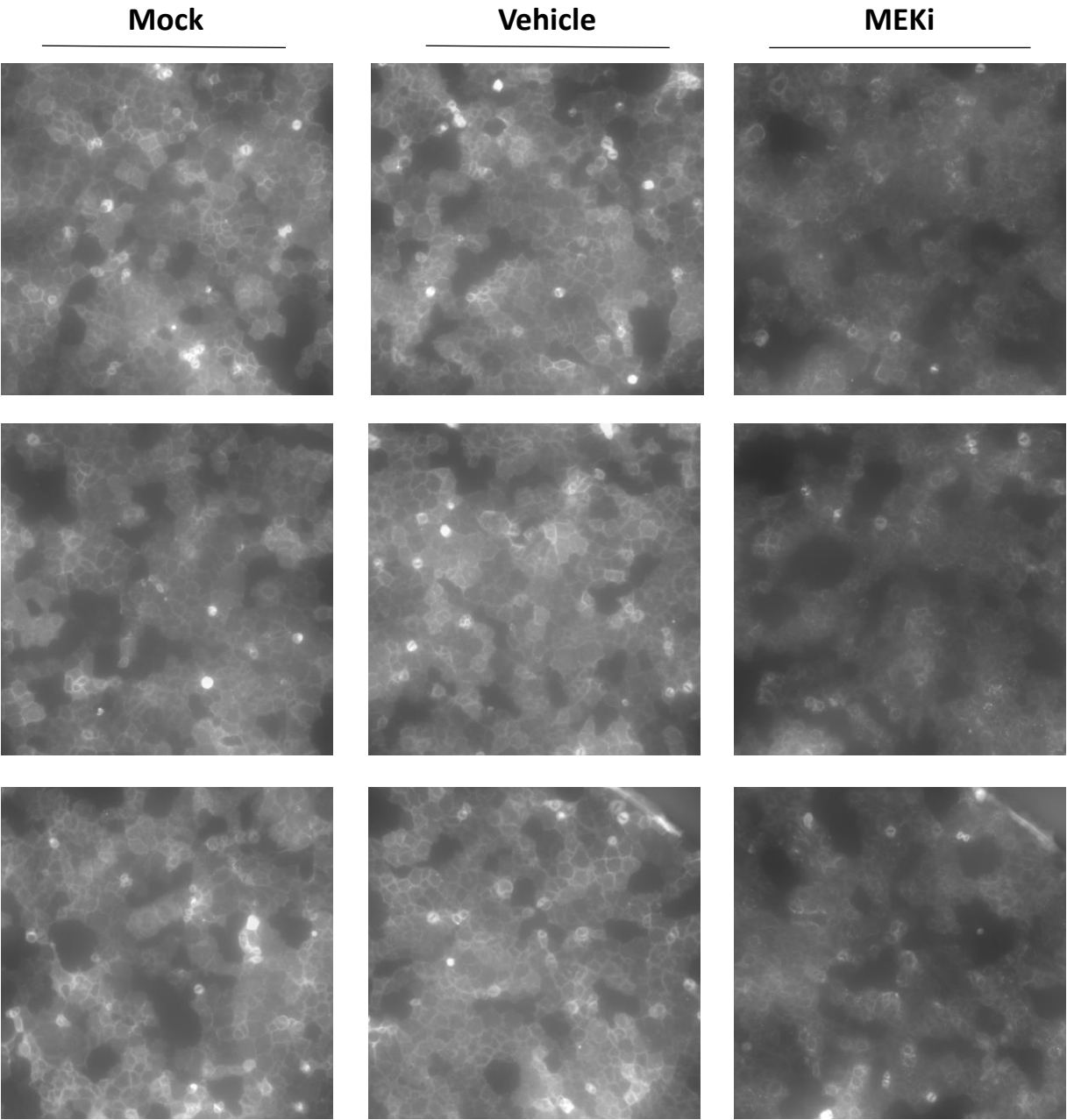

**Fig 3C**

**Vehicle**

**MEKi**

**DAPI**

**DAPI**

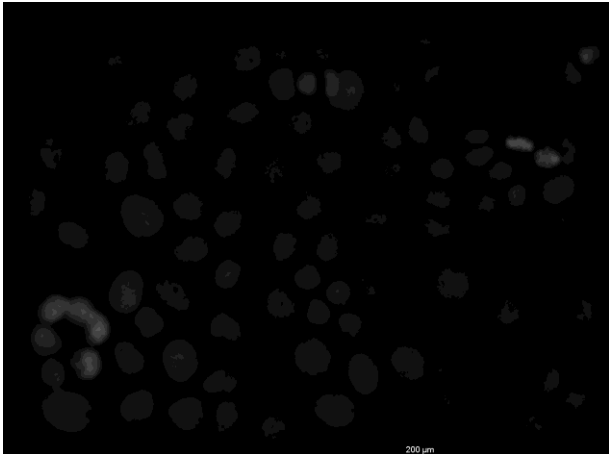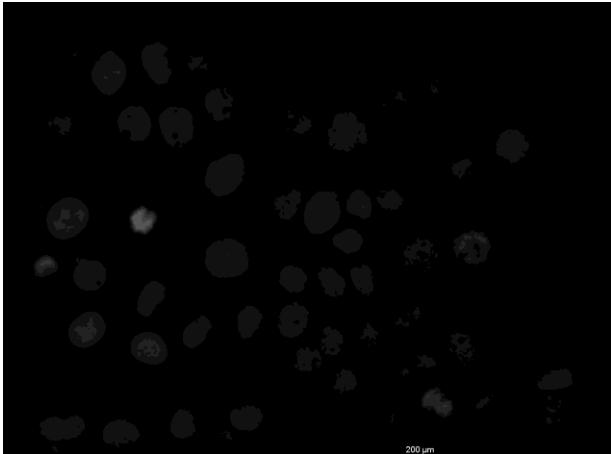

**EGFR**

**EGFR**

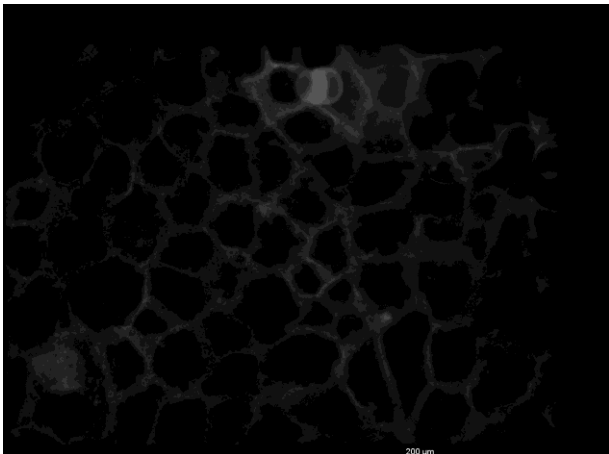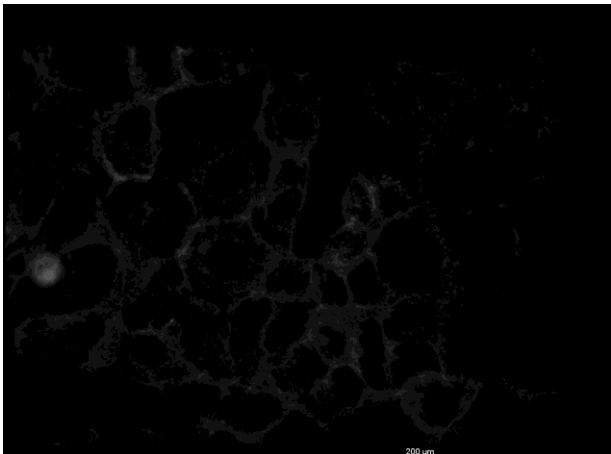

**EEA1**

**EEA1**

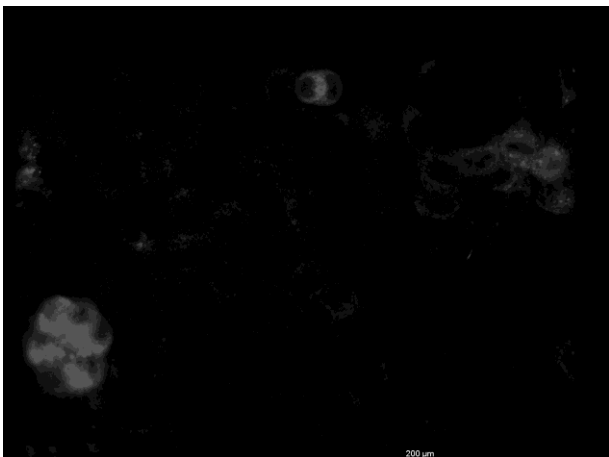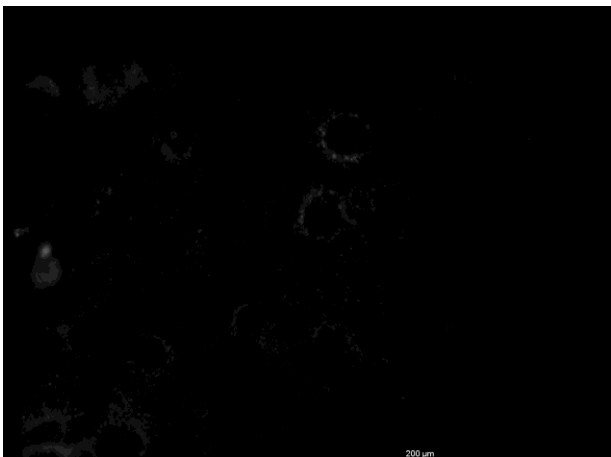

Supplement: Supplementary file 3 [file LSA-2022-01880_SdataF3.1.pdf]
