# Supplementary material for: ACE2-EGFR-MAPK signaling contributes to SARS-CoV-2 infection
Source: Life Sci Alliance. 2023 Jul 4;6(9):e202201880. doi: 10.26508/lsa.202201880 (PMC10320016; doi:10.26508/lsa.202201880)

Fig 4A

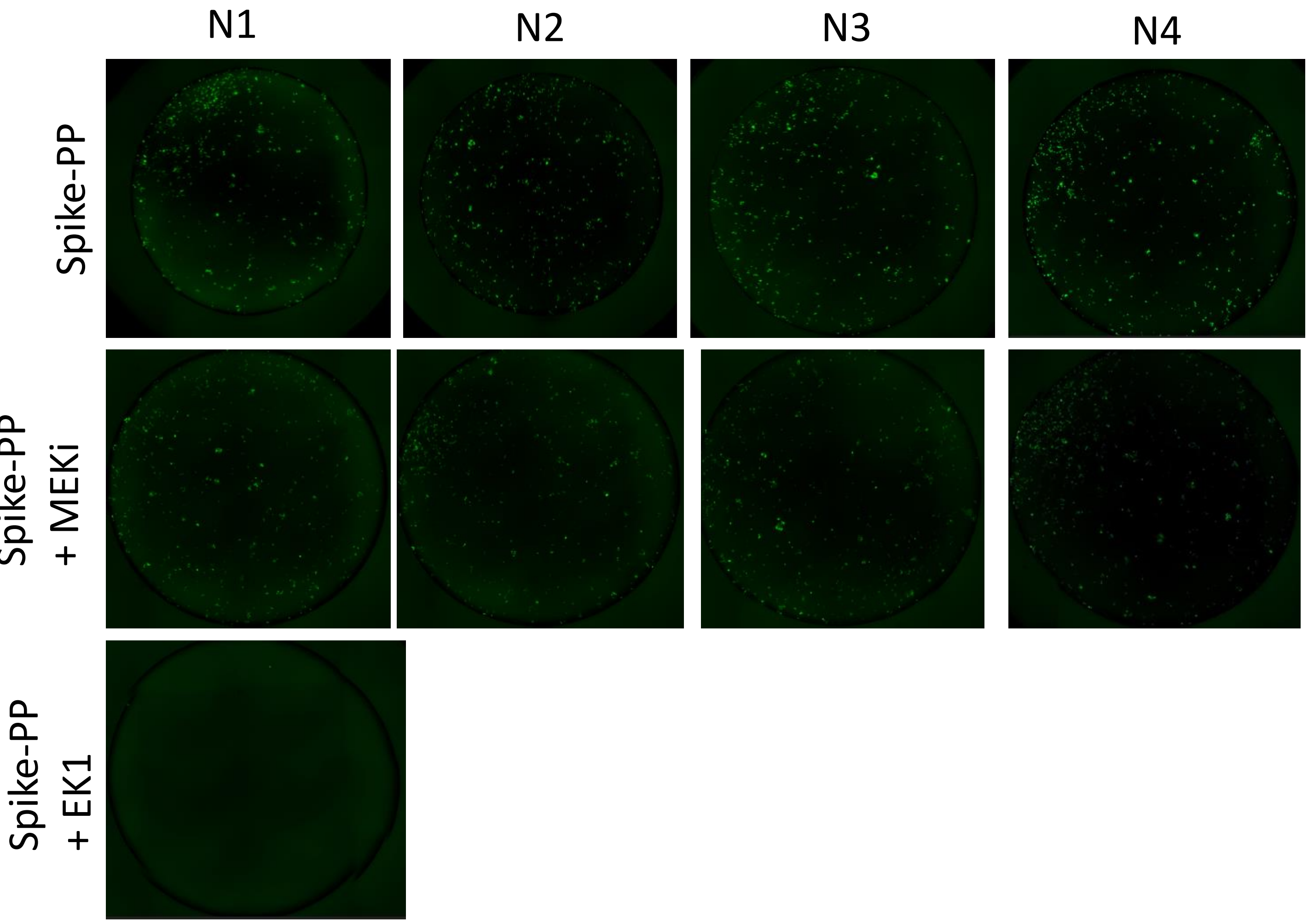

Fig 4B

VSV-Spike-PP

siRNA-NC

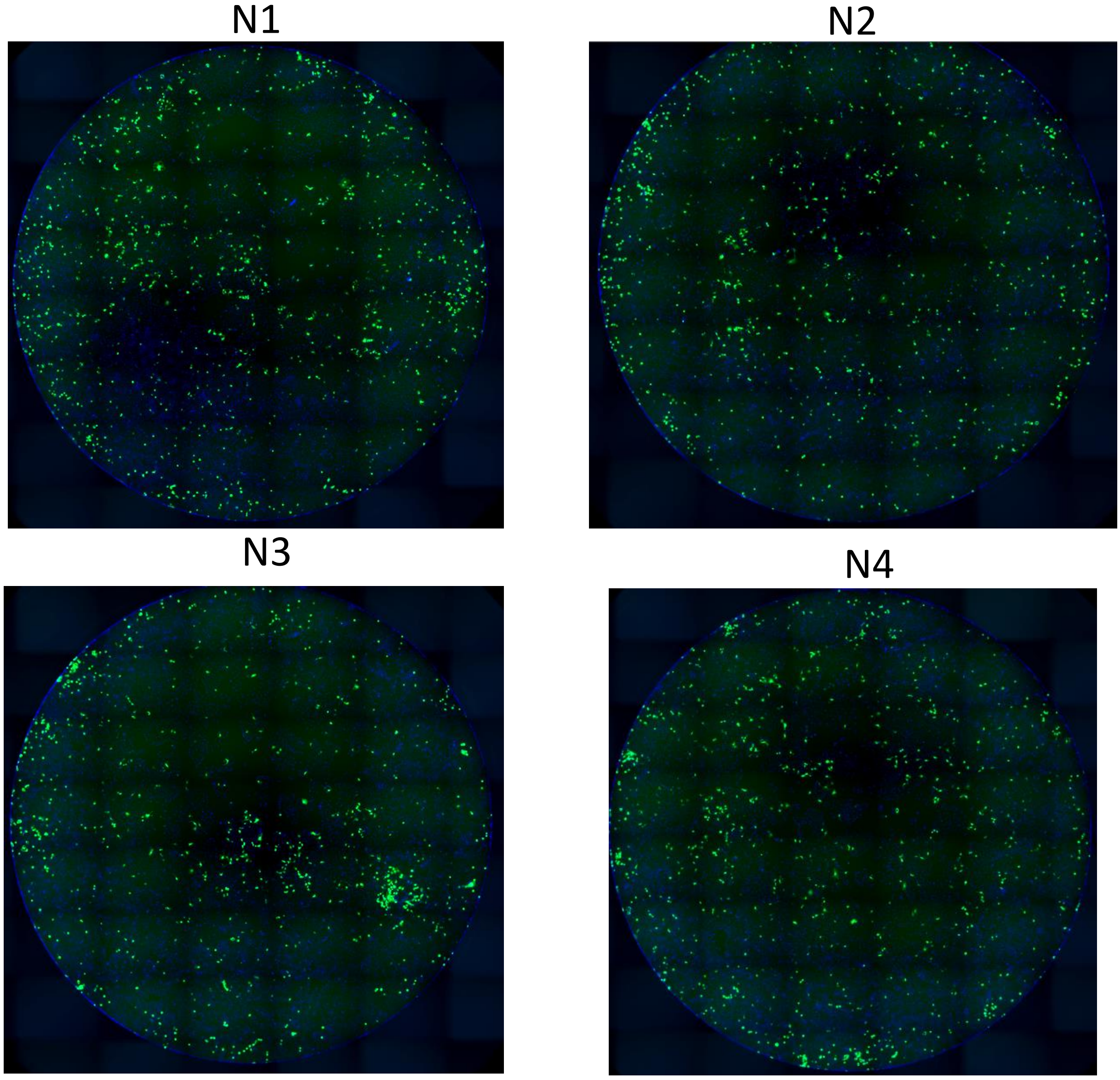

Fig 4B

VSV-Spike-PP

siRNA-ACE2

N1

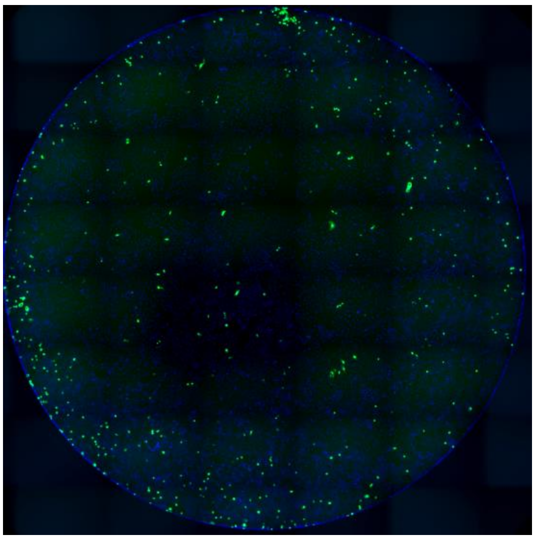

N2

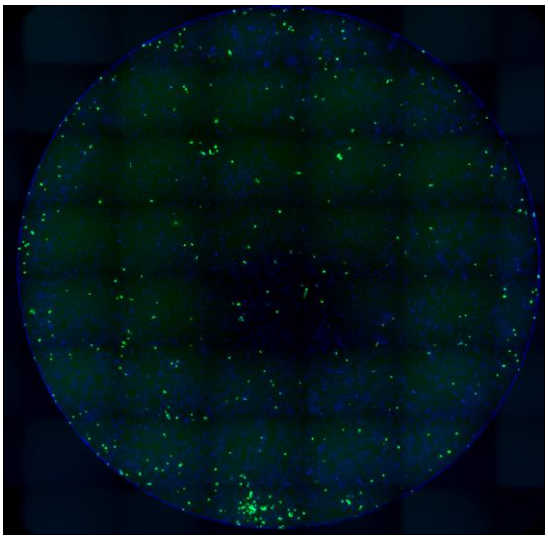

N3

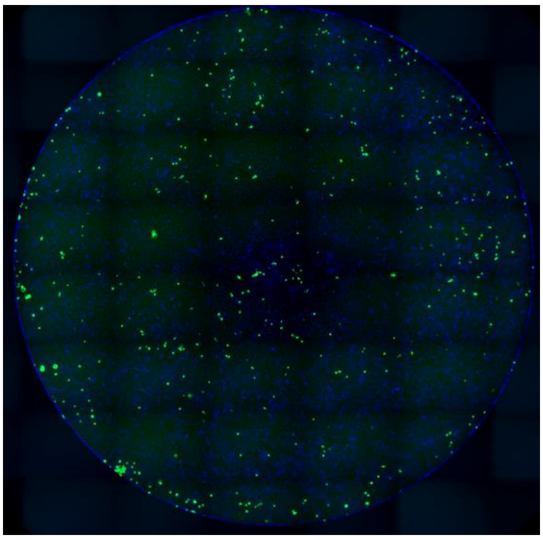

N4

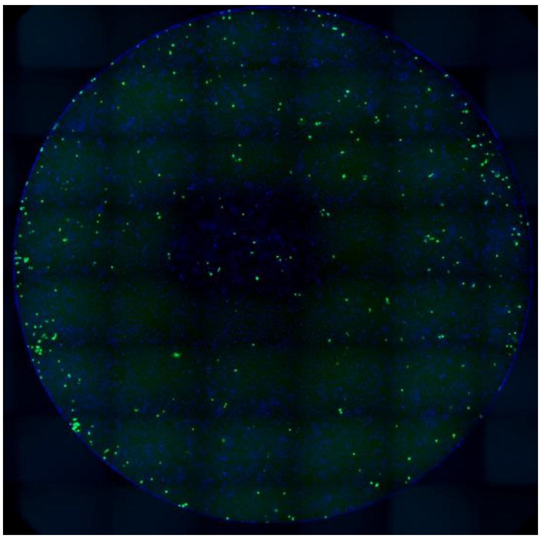

Fig 4B

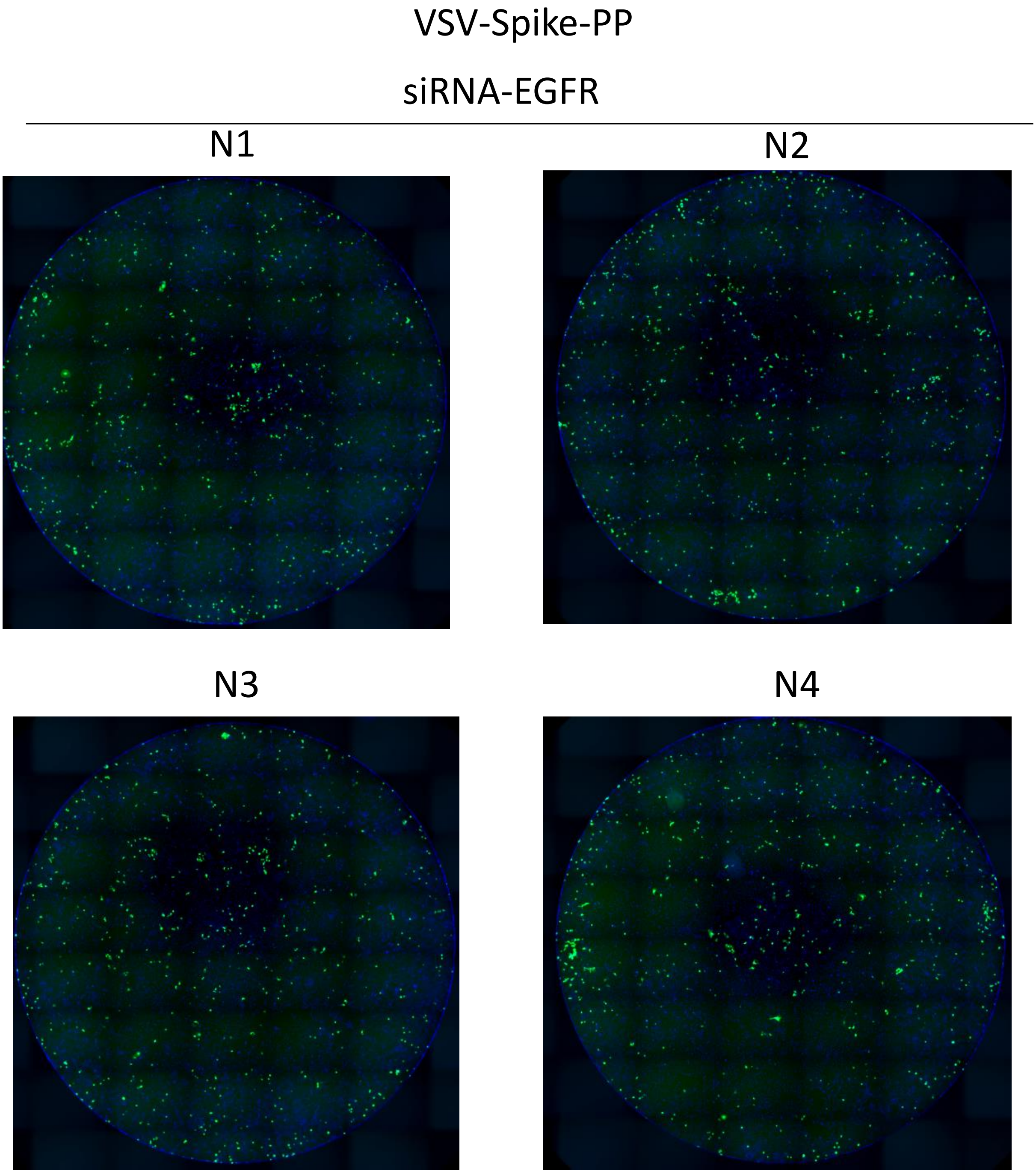

Fig 4C

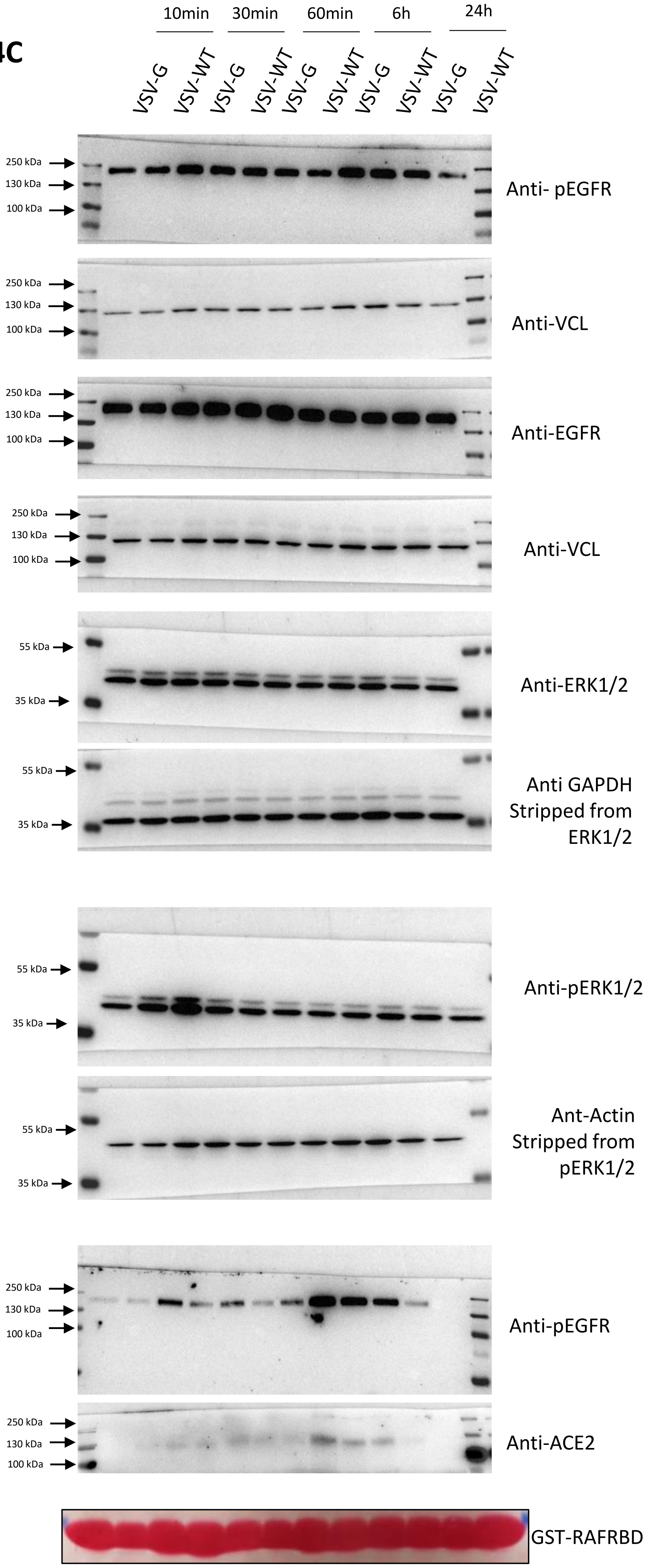
10min
30min
60min
6h
24h
VSV-G
VSV-WT
VSV-G
VSV-WT
VSV-G
VSV-WT
VSV-G
VSV-WT
VSV-G
VSV-WT
250 kDa
130 kDa
100 kDa
Anti- pEGFR
250 kDa
130 kDa
100 kDa
Anti-VCL
250 kDa
130 kDa
100 kDa
Anti-EGFR
250 kDa
130 kDa
100 kDa
Anti-VCL
55 kDa
35 kDa
Anti-ERK1/2
55 kDa
35 kDa
Anti GAPDH Stripped from ERK1/2
55 kDa
35 kDa
Anti-pERK1/2
55 kDa
35 kDa
Ant-Actin Stripped from pERK1/2
250 kDa
130 kDa
100 kDa
Anti-pEGFR
250 kDa
130 kDa
100 kDa
Anti-ACE2
GST-RAFRBD

Fig 4D

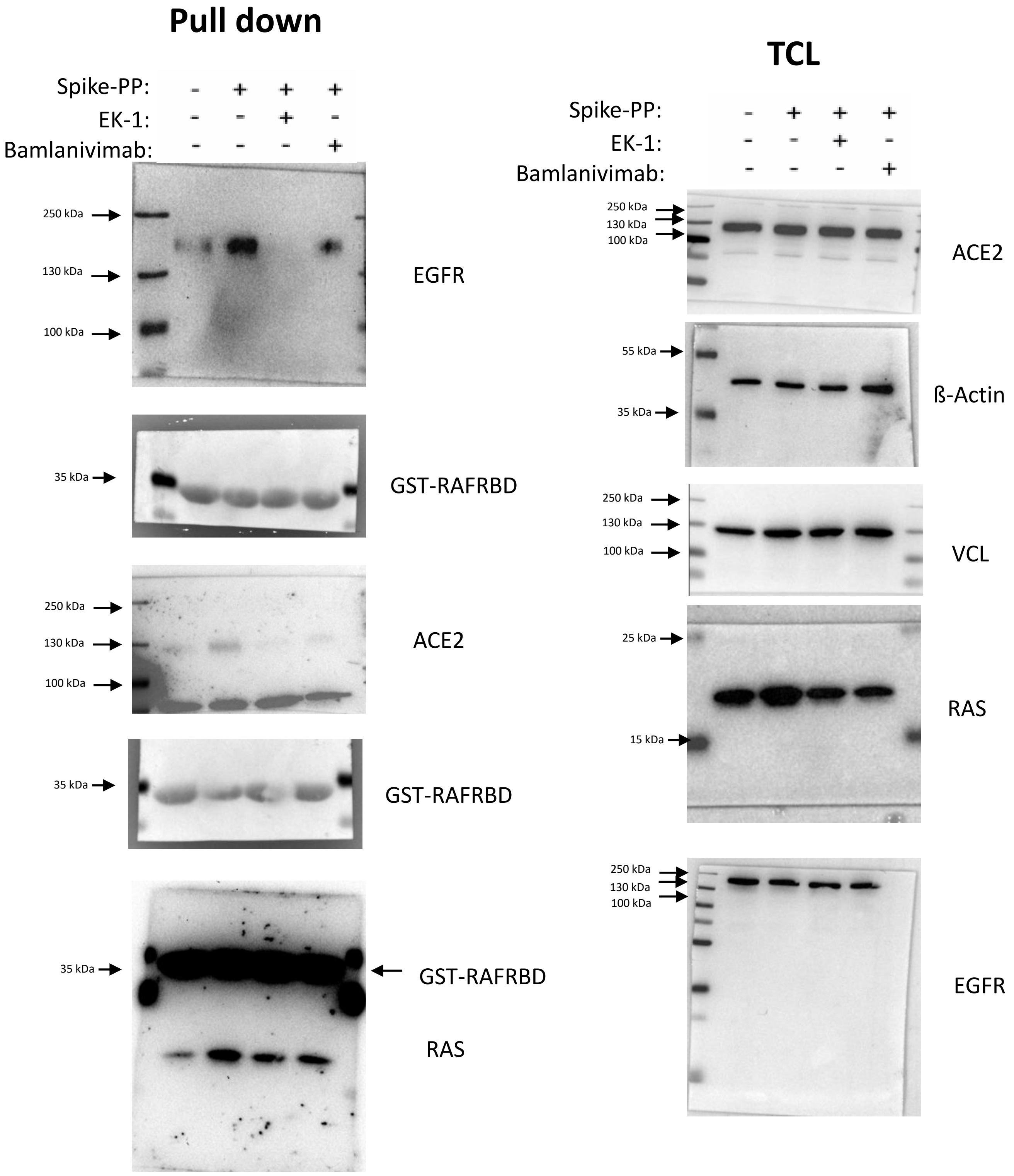
Pull down
Spike-PP: - + + +
EK-1: - - + -
Bamlanivimab: - - - +
250 kDa
130 kDa
100 kDa
EGFR
35 kDa
GST-RAFRBD
250 kDa
130 kDa
100 kDa
ACE2
35 kDa
GST-RAFRBD
35 kDa
GST-RAFRBD
RAS
TCL
Spike-PP: - + + +
EK-1: - - + -
Bamlanivimab: - - - +
250 kDa
130 kDa
100 kDa
ACE2
55 kDa
35 kDa
ß-Actin
250 kDa
130 kDa
100 kDa
VCL
25 kDa
15 kDa
RAS
250 kDa
130 kDa
100 kDa
EGFR

Fig 4E

Mock

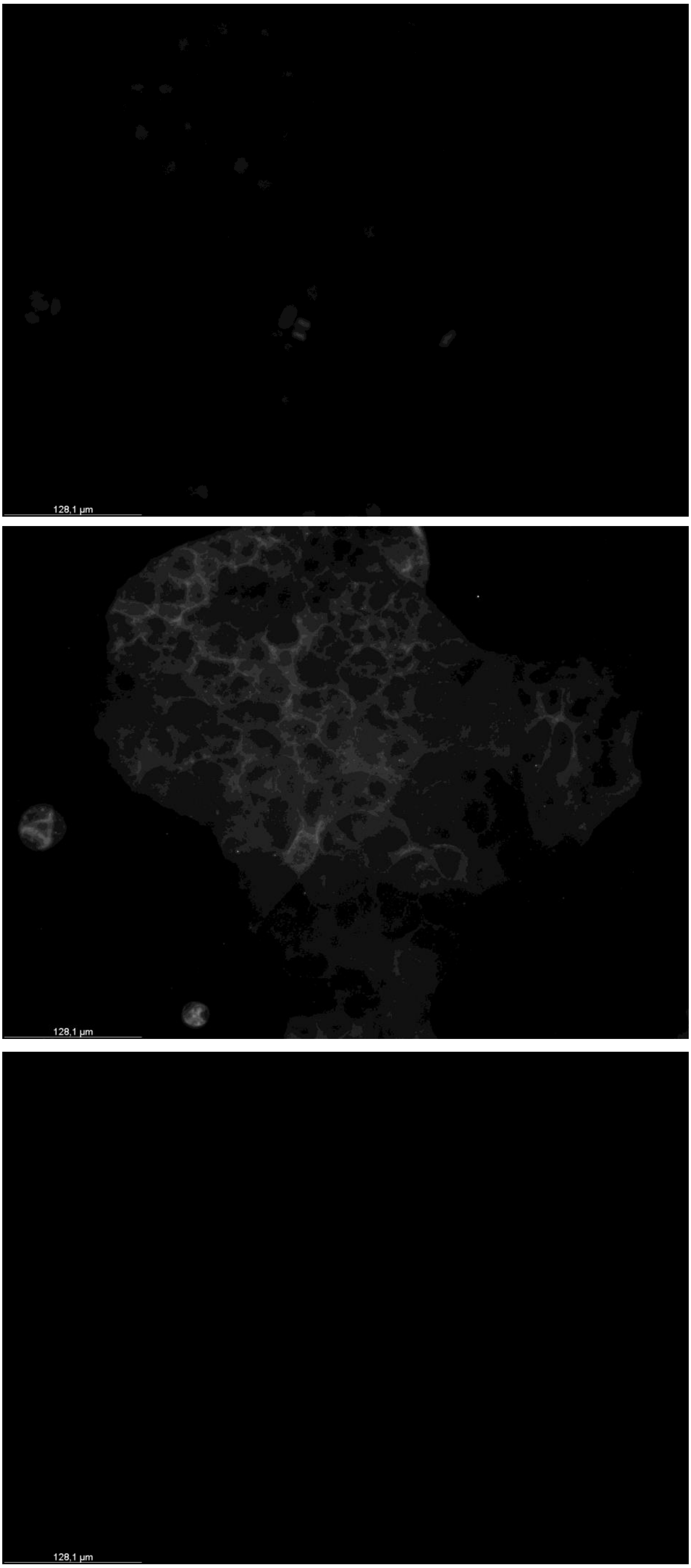

Dapi

EGFR

VSV-EYFP

Fig 4E

Spike-PP

Dapi

EGFR

VSV-EYFP

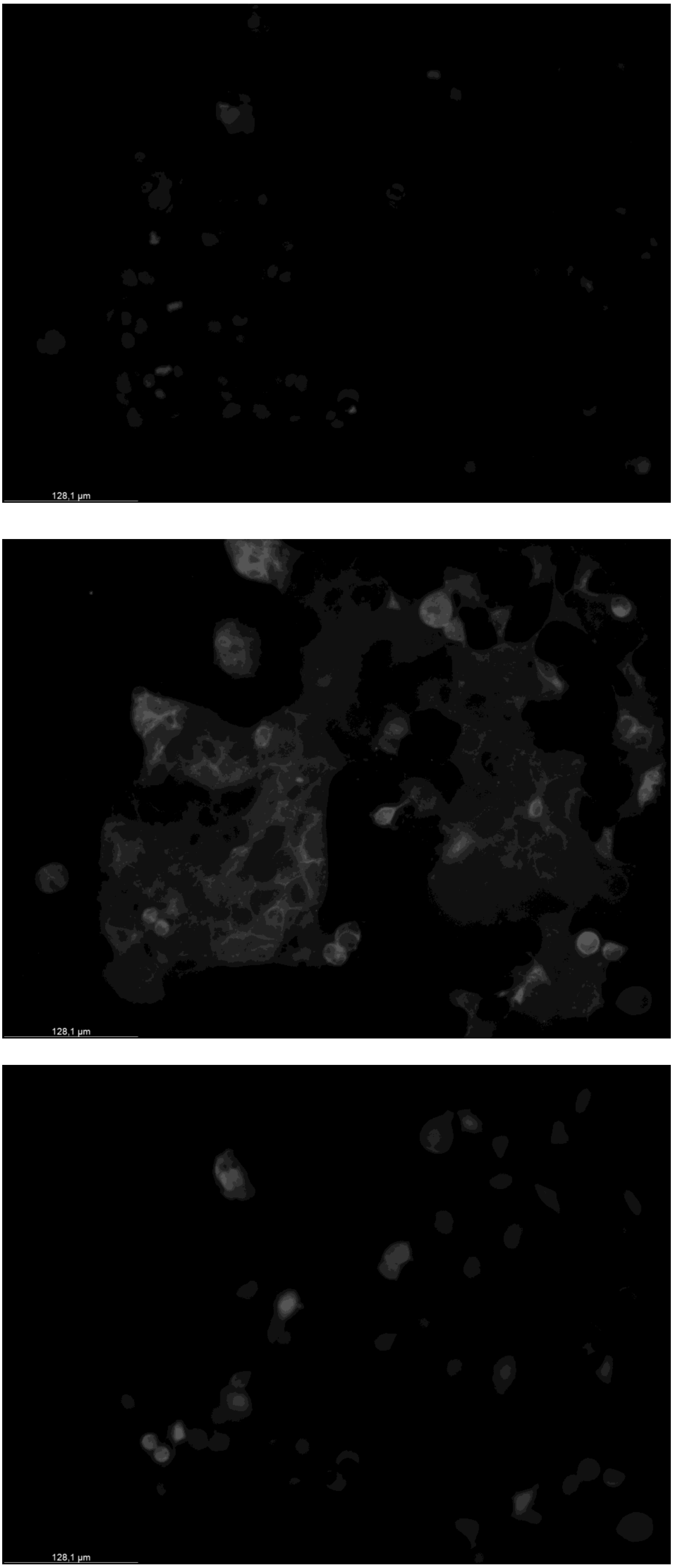

VSV-G-PP

Dapi

EGFR

VSV-EYFP

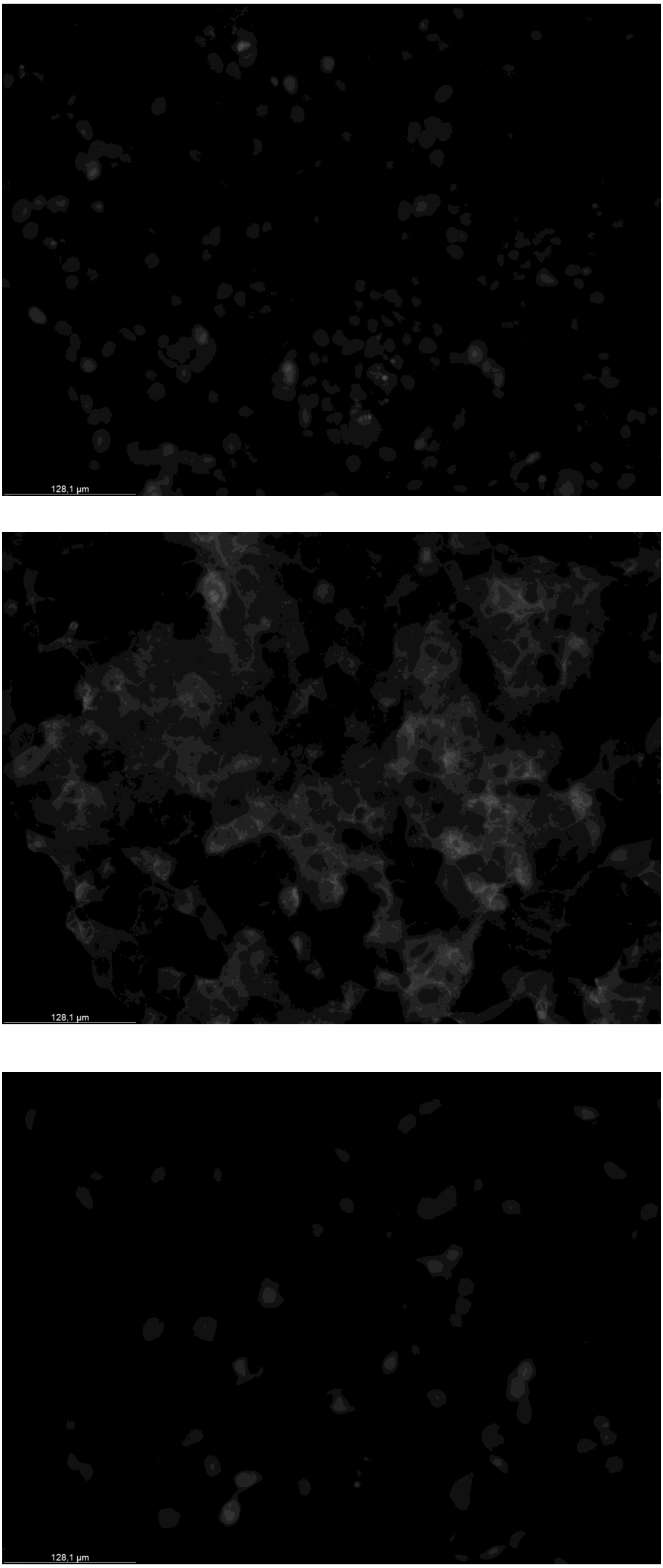

Fig 4F

Uninfected

Dapi

pEGFR

VSV-EYFP

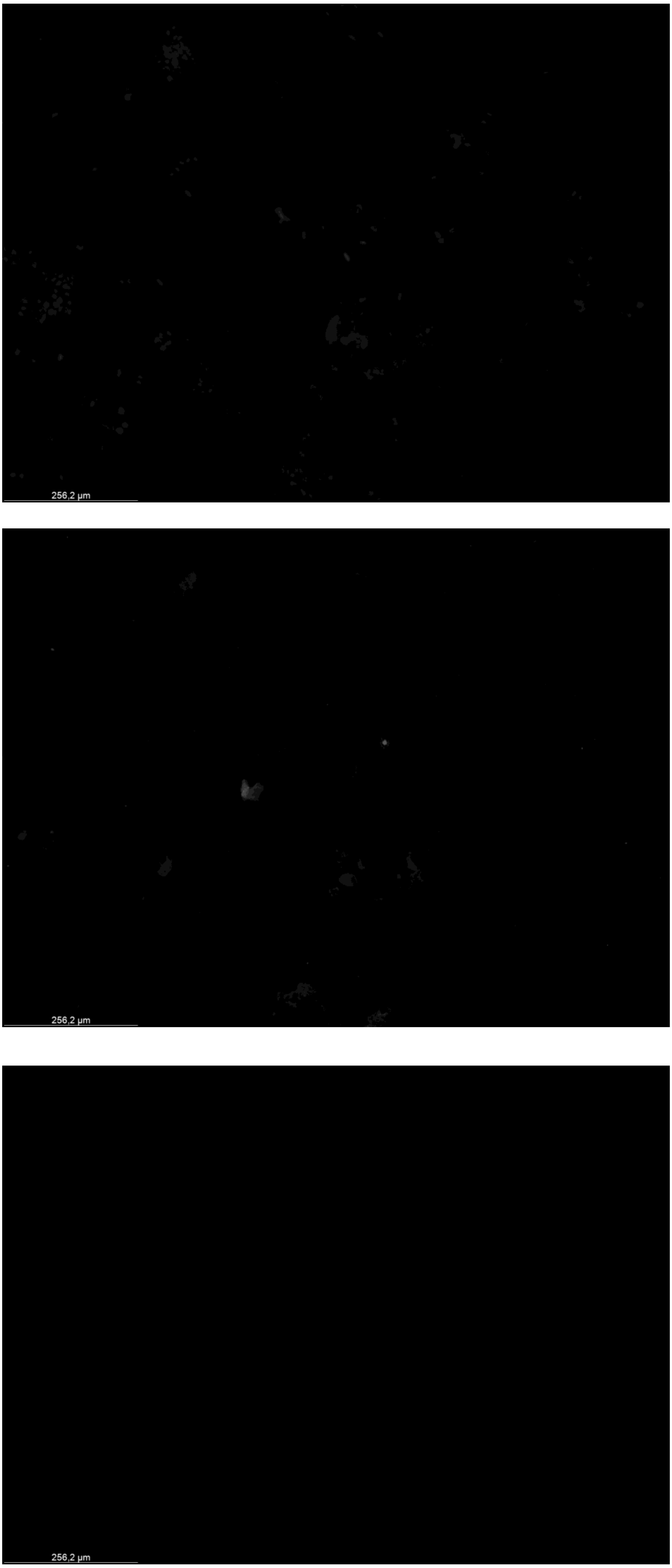

Fig 4F

VSVG-PP

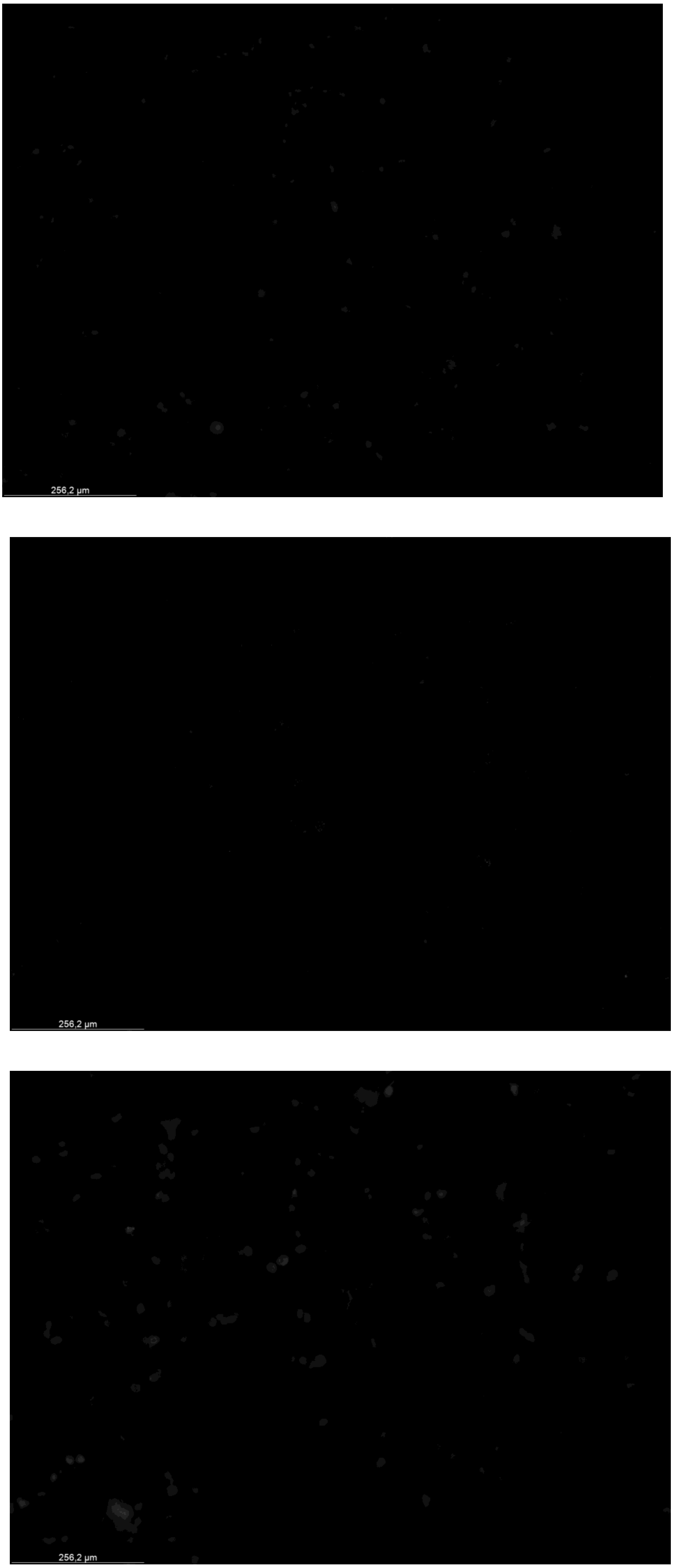

Dapi

pEGFR

VSV-EYFP

Fig 4F

Spike-PP

Merge

pEGFR

VSV-EYFP

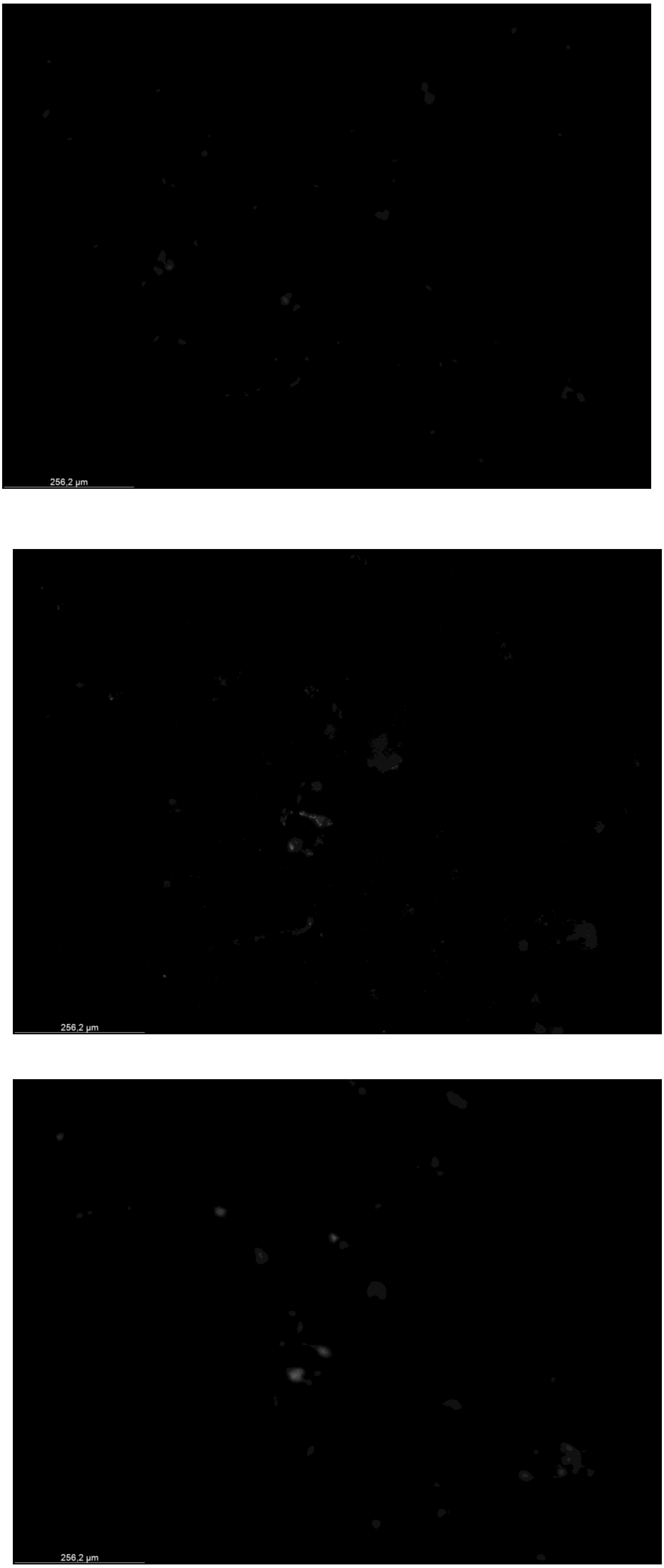

Supplement: Supplementary file 5 [file LSA-2022-01880_SdataF4.1.pdf]
