# Supplementary material for: ACE2-EGFR-MAPK signaling contributes to SARS-CoV-2 infection
Source: Life Sci Alliance. 2023 Jul 4;6(9):e202201880. doi: 10.26508/lsa.202201880 (PMC10320016; doi:10.26508/lsa.202201880)

Fig 5A

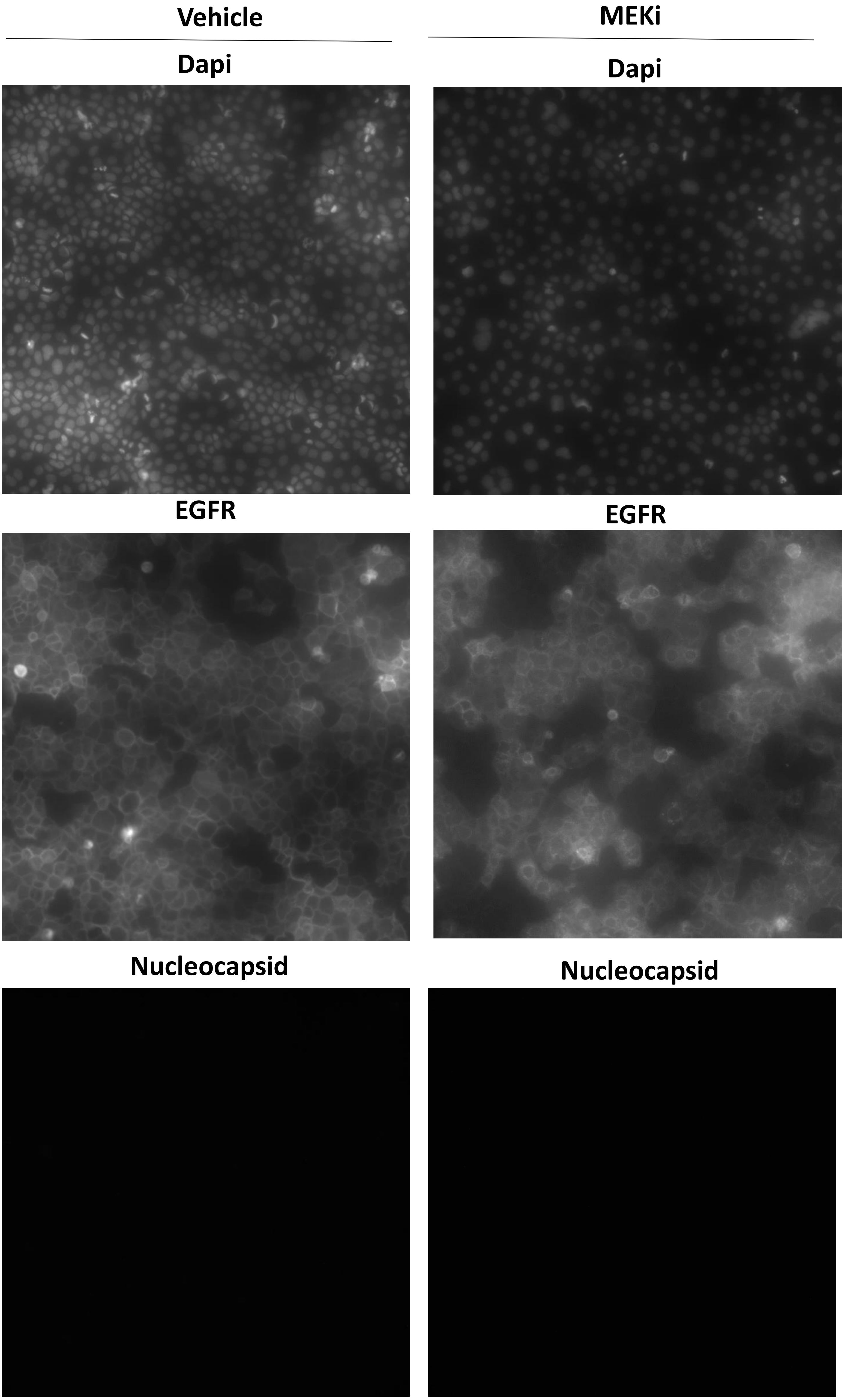
Vehicle
MEKi
Dapi
Dapi
EGFR
EGFR
Nucleocapsid
Nucleocapsid

Fig 5A

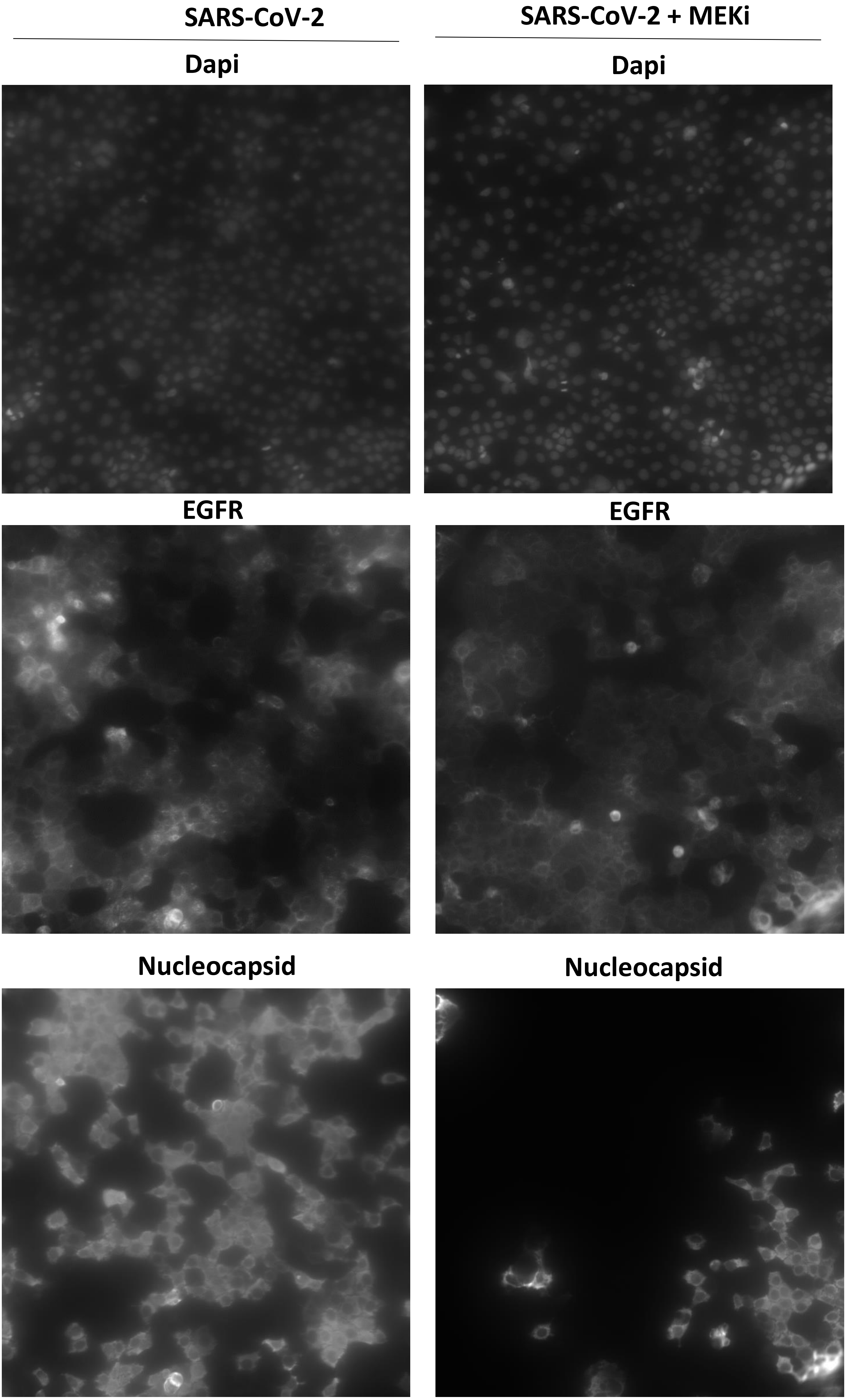
SARS-CoV-2
SARS-CoV-2 + MEKi
Dapi
Dapi
EGFR
EGFR
Nucleocapsid
Nucleocapsid

Fig 5b

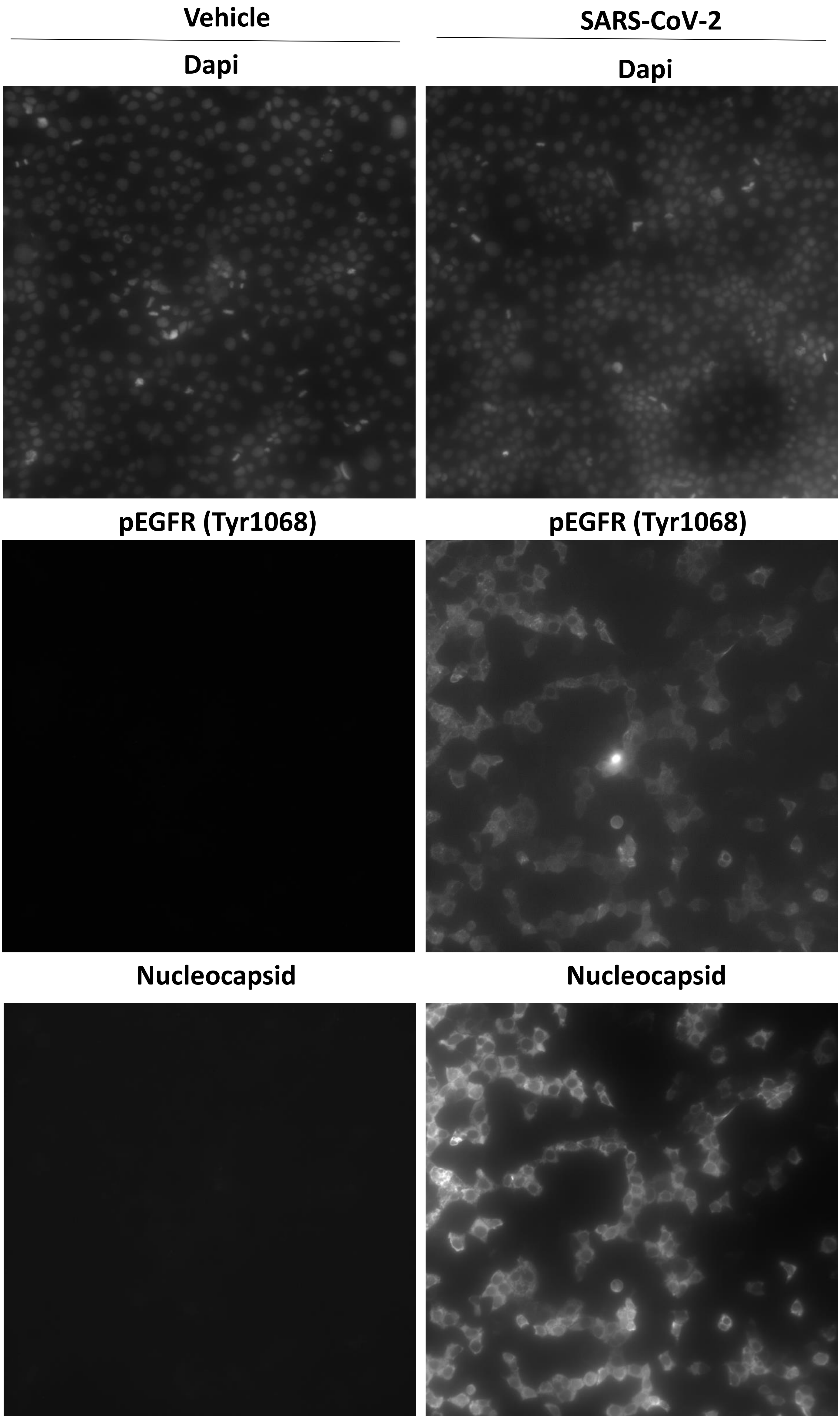
Vehicle
SARS-CoV-2
Dapi
Dapi
pEGFR (Tyr1068)
pEGFR (Tyr1068)
Nucleocapsid
Nucleocapsid

Fig 5b

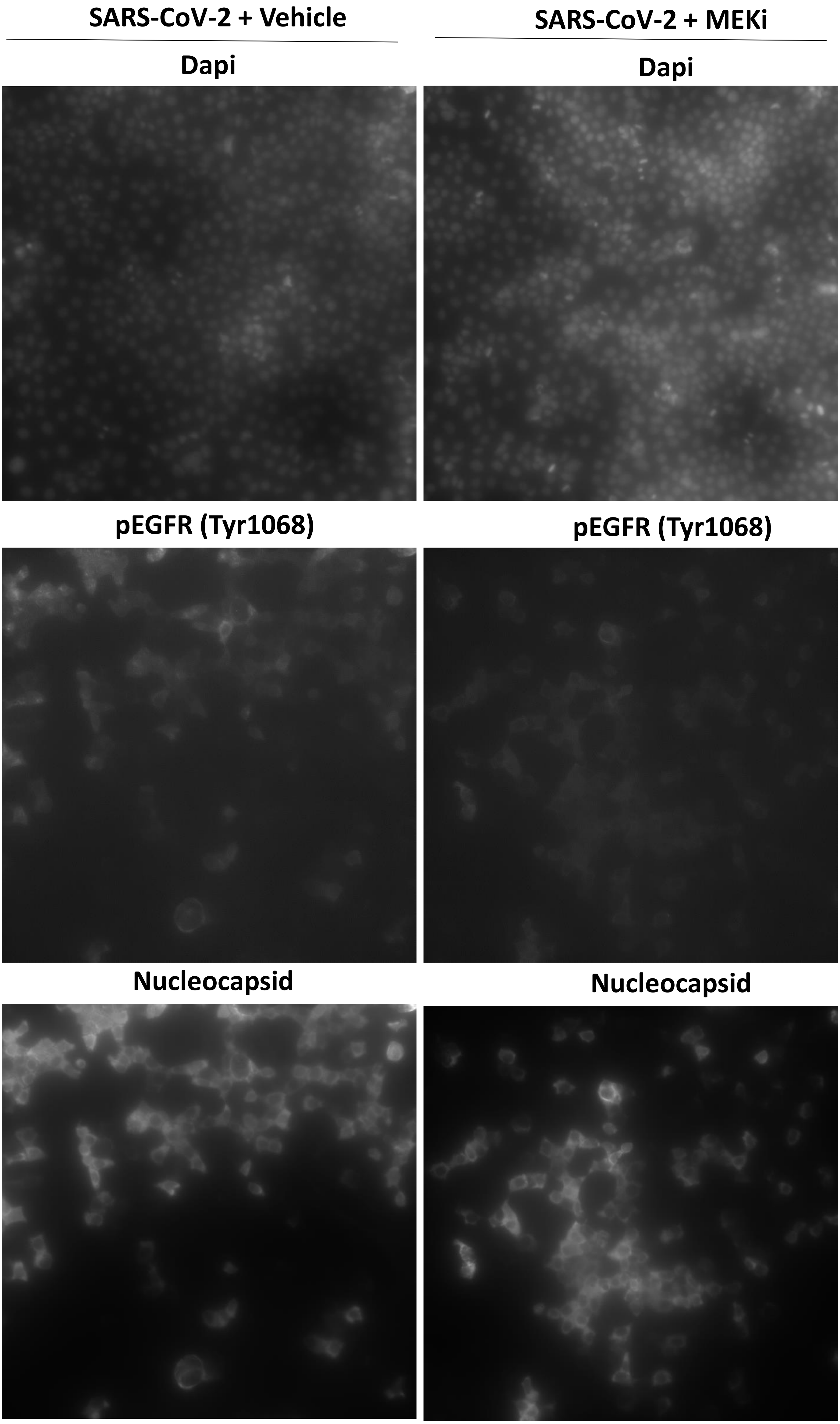

SARS-CoV-2 + Vehicle
SARS-CoV-2 + MEKi
Dapi
Dapi
pEGFR (Tyr1068)
pEGFR (Tyr1068)
Nucleocapsid
Nucleocapsid

Fig 5C

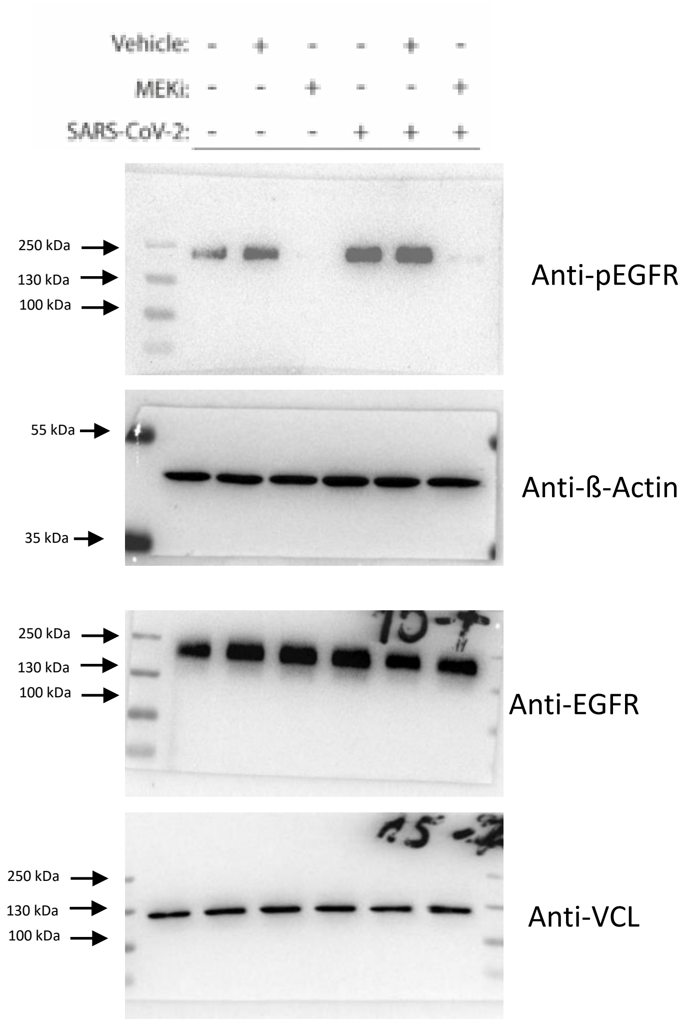
Vehicle: - + - - + -
MEKi: - - + - - +
SARS-CoV-2: - - - + + +
250 kDa
130 kDa
100 kDa
Anti-pEGFR
55 kDa
35 kDa
Anti-ß-Actin
250 kDa
130 kDa
100 kDa
Anti-EGFR
250 kDa
130 kDa
100 kDa
Anti-VCL

Fig 5D

Vehicle

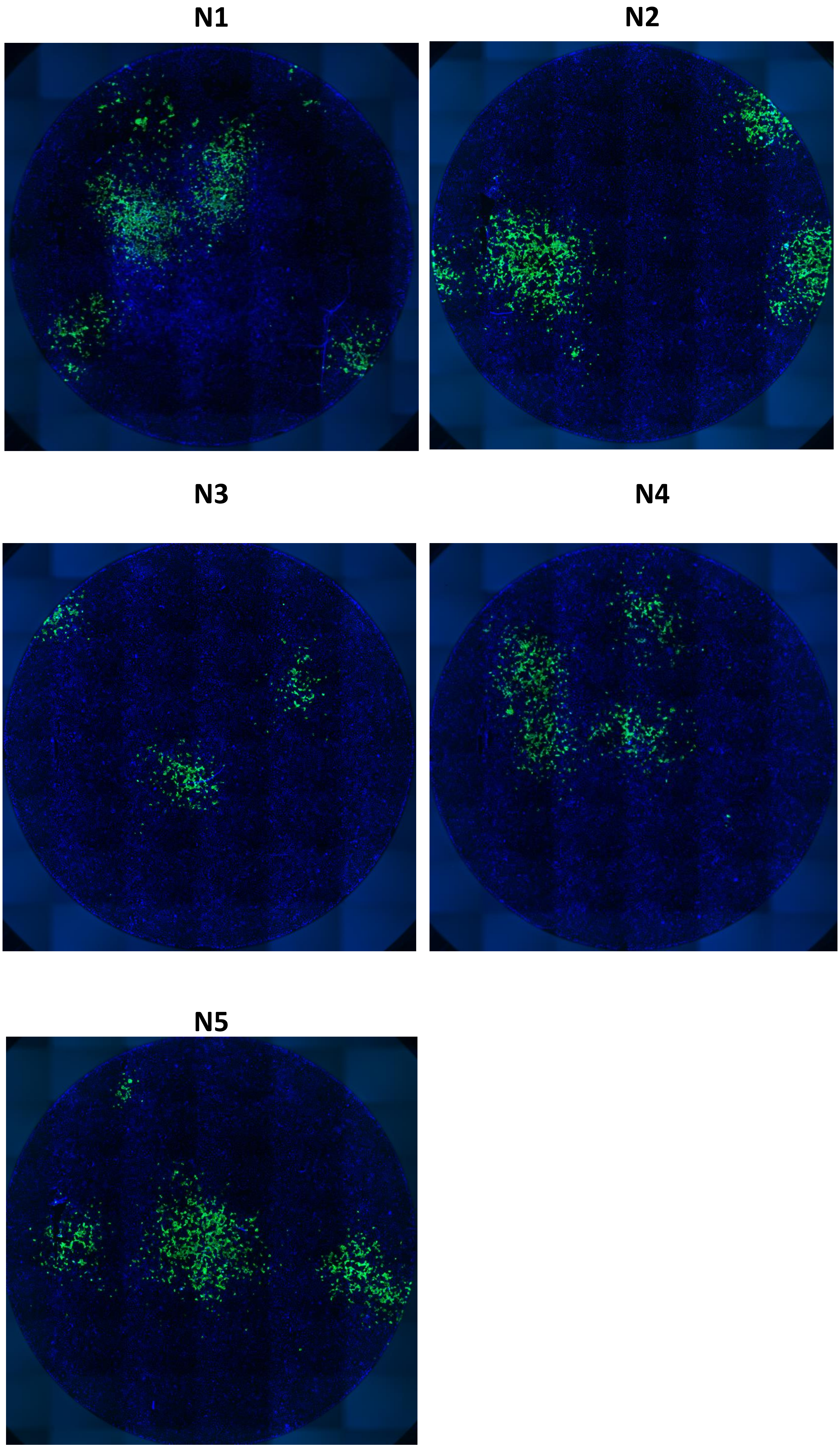

Fig 5D

MEKi

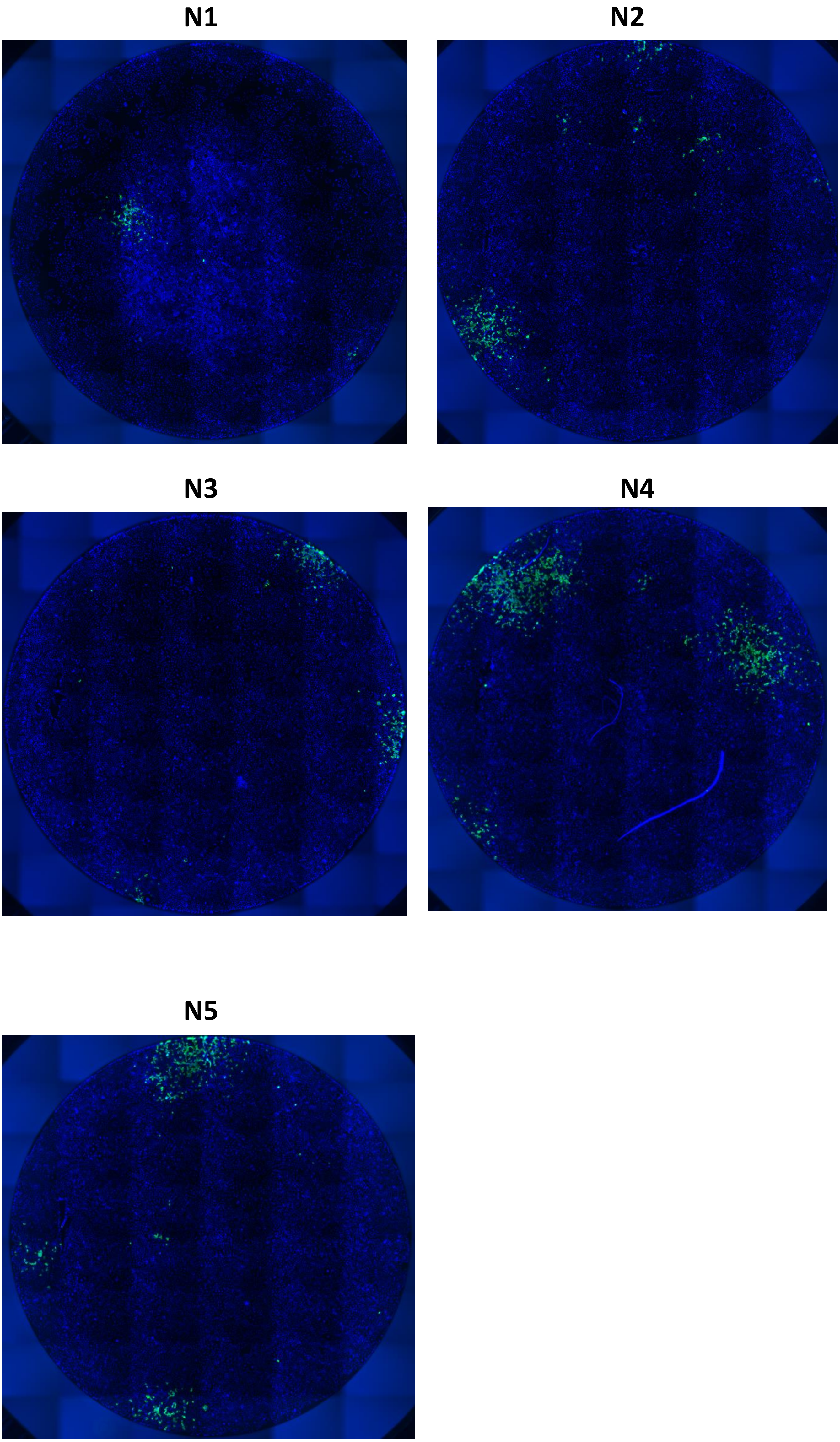

Supplement: Supplementary file 7 [file LSA-2022-01880_SdataF5.1.pdf]
